# A small molecule screen identifies novel inhibitors of mechanosensory nematocyst discharge in *Hydra*

**DOI:** 10.1038/s41598-021-99974-7

**Published:** 2021-10-18

**Authors:** Diana Hofmann, Niharika Garg, Simone Grässle, Sylvia Vanderheiden, Bruno Gideon Bergheim, Stefan Bräse, Nicole Jung, Suat Özbek

**Affiliations:** 1 Department of Molecular Evolution and Genomics, Centre for Organismal Studies, University of Heidelberg, Im Neuenheimer Feld 230, 69120 Heidelberg, Germany; 2 Karlsruhe Institute of Technology, Institute of Biological and Chemical Systems – Functional Molecular Systems (IBCS-FMS), Hermann-von-Helmholtz Platz 1, 76344 Eggenstein-Leopoldshafen, Germany; 3 Karlsruhe Institute of Technology, Institute of Organic Chemistry, Fritz-Haber-Weg 6, 76131 Karlsruhe, Germany

**Keywords:** Biological models, Experimental organisms, High-throughput screening, Drug discovery, Physiology, Chemistry

## Abstract

Cnidarians are characterized by the possession of stinging organelles, called nematocysts, which they use for prey capture and defense. Nematocyst discharge is controlled by a mechanosensory apparatus with analogies to vertebrate hair cells. Members of the transient receptor potential (TRPN) ion channel family are supposed to be involved in the transduction of the mechanical stimulus. A small molecule screen was performed to identify compounds that affect nematocyst discharge in *Hydra*. We identified several [2.2]paracyclophanes that cause inhibition of nematocyst discharge in the low micro-molar range. Further structure–activity analyses within the compound class of [2.2]paracyclophanes showed common features that are required for the inhibitory activity of the [2.2]paracyclophane core motif. This study demonstrates that *Hydra* can serve as a model for small molecule screens targeting the mechanosensory apparatus in native tissues.

## Introduction

Nematocytes of cnidarians are phylum-specific mechanosensory cells that synthesize a highly complex organelle in their cytoplasm, the nematocyst, as a secretory product^1^. Each nematocyst consists of a hollow capsule elongating into a long eversible tubule. Nematocytes respond to chemical and mechanical stimuli^2^ by a nanosecond discharge reaction during which the tubule is expelled^3,4^. The release of neurotoxins during discharge constitutes an effective mechanism of prey capture and defense. In *Hydra*, nematocytes, together with sensory and nerve cells, form a functional unit assembled in a large epithelial cell in the tentacles, the battery cell^5^. The mechanical stimulus inducing discharge is the deflection of the cnidocil at the apical end of the nematocyte. The cnidocil apparatus is composed of a central cilium surrounded by a semicircle of stereocilia^6^. Mechanoreception involves the gating of a transmembrane ion channel, which results in a receptor potential due to cation influx^7,8^.

The superfamily of transient receptor potential (TRP) proteins, which comprises seven subfamilies (TRPA, TRPC, TRPM, TRPML, TRPN, TRPP, and TRPV), are non-selective ion channels enabling animals to sense a variety of environmental stimuli, such as light, odors, temperature, and mechanical forces^9–12^. Their molecular architecture is characterized by six transmembrane domains and, in most subfamilies, the possession of *N*-terminal ankyrin repeats arranged in a tandem array. The ankyrin tail, which is longest in TRPN channels (29 repeats), is hypothesized to mediate the opening of the channel pore by acting as a molecular “gating spring”^13,14^.

We have recently shown that members of the TRPN channel subfamily are expressed in developing nematocytes and localized at the mechanosensory cnidocil apparatus of *Hydra magnipapillata*^15^. The four TRPN paralogues (TRPN1-4) found in the *Hydra* genome were mostly localized at the central cilium of the cnidocil, indicating a prominent role in the mechanosensory discharge process. TRPN4, which showed a membrane staining in developing and mature nematocysts, is proposed to have a specialized function in capsule morphogenesis^15^.

Up to date, most TRPN channels were identified in invertebrate taxa^16^. The *Drosophila* homolog, NOMPC, has been studied extensively and functions in touch sensation of larvae^12^ and hearing of larvae and adult flies^17^. Recently, it has been possible to form functional mechanotransduction channels by heterologous expression of NOMPC, facilitating functional studies^18^. In spite of this, analyses of TRPN channels in native tissues are still hampered by a lack of specific pharmacological tools.

This study aimed to establish a small molecule compound screen that enables the identification of new compound classes with the potential to affect the TRPN channel function in the cnidarian *Hydra magnipapillata*, based on nematocyst discharge. As the inhibition of TRPN channels by small molecules is not well understood so far, a straightforward strategy to gain information on potential candidates within random chemical compound libraries was envisaged. Therefore, we tested 700 small molecules, gained as a random selection of diverse compounds from an academic compound collection, for their capacity to inhibit nematocyst discharge in living Hydras. Out of these, we have been able to identify members of the [2.2]paracyclophane family as a novel group of potential TRPN inhibitors, which might also be functional in related mechanosensory channels of mammals, as the TRPA1 ion channel. The compound class of [2.2]paracyclophanes, showing planar chirality, if substitutions are introduced to the [2.2]paracyclophane core, is well characterized as a chemical catalyst and in materials science applications^19^, but only a few studies have highlighted [2.2]paracyclophanes with respect to their potential biological activities^20,21^.

## Results

### Initial prey capture screen

**Figure 1:**
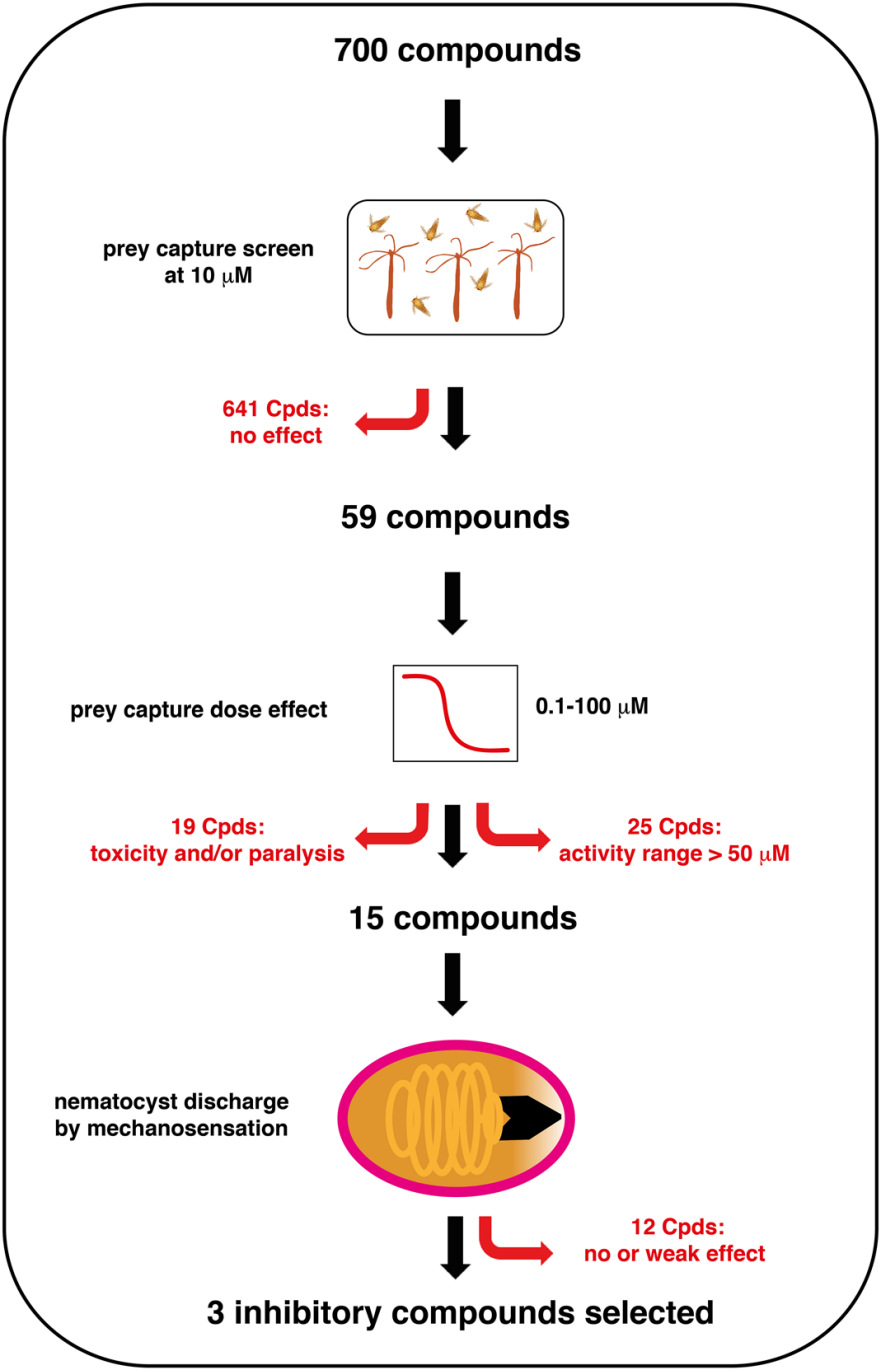
Schematic overview of inhibitor screen. A collection of 700 small molecule compounds was screened for nematocyte discharge inhibition in *Hydra* by three consecutive stages. In the initial prey capture screen, a majority of compounds lacking any inhibitory effect at 10 µM were excluded. The remaining 59 compounds were screened for their activity range in the prey capture screen, leaving 15 positive candidates with half-maximal inhibition at 50 µM or lower. Compounds inducing high toxicity (11 compounds) or paralysis (8 compounds) during long-term exposition were excluded at this stage. Toxicity was evaluated using 5 categories: 0, no visible toxic effects; 1, slightly retracted tentacles; 2, pronounced tentacle retraction; 3, complete tentacle retraction and body contraction; 4, as in 3 and animals partly dissociated; 5, animals completely dissociated and/or dead. In the last stage, 3 compounds showing full discharge inhibition for a purely mechanical stimulus and half-maximal inhibition at 5 µM or lower were selected as the most potent candidate substances.

**Figure 2:**
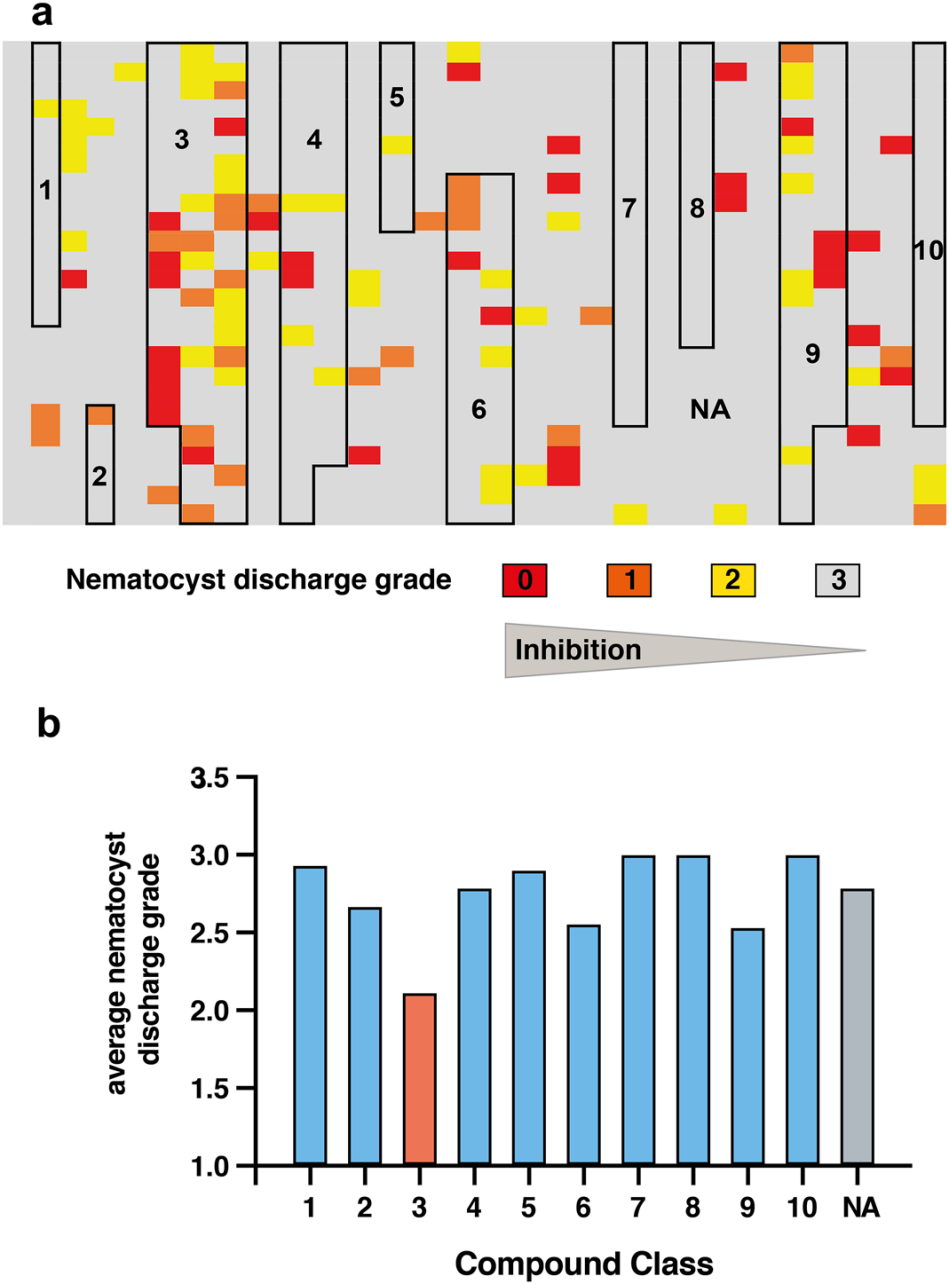
(**a**) Heat map of compound library screen including 700 compounds. 10 selected compound classes are highlighted as indicated. Colors indicate grades of effective nematocyst discharge derived from prey capture efficiency as recorded in the primary screen. Grey, grade 3 (prey capture as in control); yellow, grade 2; orange, grade 1; red, grade 0 (full prey capture inhibition). The applied grades are further detailed in methods. Compound classes are indicated with boxes and numbers. Detailed data are given in Supplementary Table S1 online. (**b**) Average nematocyst discharge grade for tested compound classes applying levels 0–3 obtained from the primary screen (no influence on prey capture equals 3). 1 = pyrazoles, 2 = triazenes, 3 = [2.2]paracyclophanes (orange), 4 = coumarins, 5 = sugars, 6 = (per)fluorinated compounds, 7 = piperazines, 8 = steroids, 9 = azo-compounds, 10 = indole derivatives. NA (not assigned) = compound with no assignment to a certain compound class (grey).

In the first stage of our screen for nematocyst discharge inhibitors, we used steady-state adult polyps of *Hydra magnipapillata* exposed to the small molecules at a concentration of 10 µM in 24-well plates (see Fig. 1 for a schematic overview of the inhibitor screen). As a positive control, Streptomycin (STM), a known inhibitor of mechanically induced discharge of *Hydra* nematocytes, was applied at 100 µM^22^. Five animals per well were incubated for 30 min in *Hydra* medium (HM) containing the compounds before freshly hatched *Artemia* were added to the wells. Prey capture was scored after 10 min by recording the ratio of animals showing a reduced killing of prey as compared to control animals incubated in HM. The effective nematocyst discharge grade was recorded using a scale from 0 (full prey capture inhibition in all animals) to 3 (normal prey capture in all animals as in control), allowing a fast evaluation of the 700 compounds via a heat map analysis (Fig. 2). In a parallel setting, we monitored toxic effects during the initial prey capture screen and inspected animals during a 48-h exposure time to the small molecules tested. Compounds showing toxic effects were not removed from the screen at this stage to be able to re-evaluate toxicity within a broader concentration range during the second screening stage.

After the first screening stage, 59 compounds were found to be active in prey capture inhibition (Fig. 1). The tested compounds were further classified according to their core structure to generate an indication of compound classes that offer the highest potential with respect to prey capture inhibition. This structure-based sorting of the tested compounds resulted in identifying 10 compound classes with more than 5 representatives in the primary assay (Fig. 2a, Supplementary Table S1 online). Altogether 285 compounds could be assigned to one of 10 compound classes while 415 compounds could not be sorted to a certain class (or belonged to a group with < 6 representatives). A comparison of the compound classes was achieved by calculating the average nematocyst discharge grade applying the levels 0–3 used for the primary screen (no influence on prey capture equals 3). It was found that the unclassified compounds have an average nematocyst discharge efficiency score of 2.8 (NA in Fig. 2b), while triazenes (class 2, efficiency score of 2.7), [2.2]paracyclophanes (class 3, efficiency score of 2.1), (per)fluorinated compounds (class 6, efficiency score of 2.6), and azo compounds (class 9, efficiency score of 2.5) used in this study showed a higher inhibitory effect (Fig. 2b). Pyrazoles (class 1), coumarins (class 4), and sugars (class 5) showed either the same or less activity than the unclassified compounds. Compounds belonging to the classes of piperazines (class 7), steroids (class 8), and indole-derivatives (class 10) did not effect prey capture. In summary, the results of this comparison indicated that, already after the primary assay, [2.2]paracyclophanes might serve as an interesting compound class for the inhibition of nematocyst discharge.

### Dose–response studies for selected inhibitory compounds

All 59 compounds that showed an inhibitory effect in the primary screen (red and orange color, Fig. 2a) were tested with respect to their dose–response activity independent of their assignment to a certain compound class. For this purpose, a more refined prey capture assessment was performed within a concentration range of tested compounds between 0.1 and 100 µM. Substances showing high toxicity (grade 3 or higher) at compound concentrations of 25 µM or higher were excluded at this stage. Animals showing a significant reduction in prey capture efficiency were challenged additionally by touching with a glass pipette tip to rule out false-positive effects by systemic paralysis. *Hydra* polyps normally react to this stimulus by immediate longitudinal contraction and retraction of tentacles. 8 compounds were found to induce paralysis. In addition, as we aimed to reach an equal or more effective dose compared to STM (half-maximal inhibition at ~ 10 µM)^22^ we excluded compounds that showed a half-maximal inhibition at 50 µM or higher. This second screening stage resulted in 15 promising candidate substances with [2.2]paracyclophanes as the most active compound class (5 derivatives) (Fig. 1, Supplementary Fig. S1a online).

**Figure 3:**
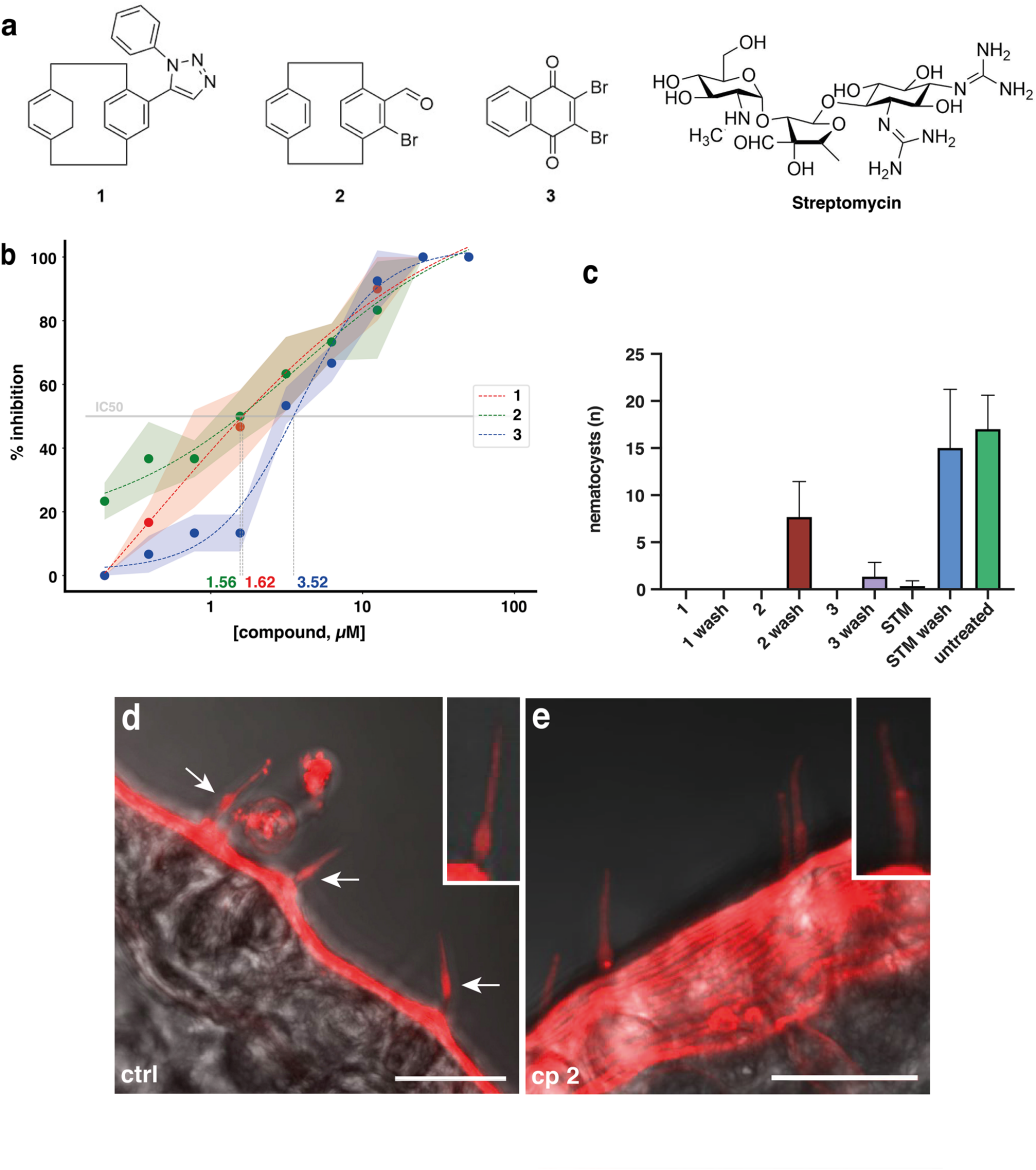
Detailed analysis of nematocyst discharge inhibition for three candidate molecules. (**a**) Structures of compounds **1**, **2,** and **3**. (**b**) Prey capture inhibition at different concentrations of selected small molecules. Animals were treated in triplicates with the indicated concentration of the compounds for 30 min. Nematocyst discharge was recorded by counting the fraction of artemia (out of ten) caught by each polyp. The inhibitor concentration is shown on the log scale. Colored areas correspond to standard deviation. IC50 values were determined by fitting sigmoid curves to the average inhibition (dashed lines) and solving them for 50% inhibition. (**c**) Reversibility assay of mechanosensitive nematocyst discharge was tested by recording discharge in the presence of each selected compound at 12.5 µM and again immediately after compound removal (wash). Nematocyst discharge was evaluated by counting the number of attached stenoteles after mechanical stimulus with a gelatin-covered fishing line. Error bars represent standard deviations from triplicate experiments. STM, Streptomycin. (**d**) Immunostaining of Hydra whole-mounts with a pan-TRPN antibody shows a prominent signal at the base of cnidocils (white arrows) extending from battery cells of the tentacle surface. Control animals were treated with DMSO. (**e**) Treatment with compound **2** for 2 h leads to a loss of the signal probably due to the relocalization of TRPN in the battery cells. Scale bars = 10 µm. The insets show exemplary cnidocils at higher magnification. Images were acquired with a Nikon A1 confocal microscope using imaging software NIS Elements (AR 45.51.01).

We next analyzed the capacity of the 15 selected compounds for blocking mechanical nematocyte discharge by touching each tentacle of treated animals with the tip of a clean glass pipette. Nematocyte discharge in satiated polyps is triggered by a combination of mechanical and chemical stimuli as elicited by prey organisms. After prolonged starvation (7 days), discharge can also be induced via a purely mechanical stimulus^22^. We evaluated discharge by counting the average number of animals out of ten, which stayed attached to the pipette surface after short tentacle contact (1–2 s), indicating nematocyst discharge. For this assay, we applied compound concentrations of 25 and 50 µM, respectively (Supplementary Fig. S1b online). Three small molecules (compounds **1**, **2,** and **3**, Fig. 3a) showing a full inhibitory effect on the mechanical sensory component of nematocyte discharge and exhibiting half-maximal inhibition below 5 µM (Supplementary Fig. S1a,b, online) were finally selected. The dose–response curves and IC50 values of these compounds are shown in Fig. 3b. We next tested the reversibility of the mechanosensory discharge inhibition by application of each selected compound at 12.5 µM (Fig. 3c). To have a better quantitative readout for nematocyst discharge, we used a gelatin-covered fishing line for touching the tentacles of the animals and counted the large stenotele type of nematocysts attached to the fishing line using light microscopy. Reversibility of discharge inhibition by the three compounds was examined by replacing the medium with fresh HM and immediate repetition of the mechanical stimulus. In this assay, the inhibitory effect of compound **2** was largely reversible, while animals treated with the other compounds showed no or very little recovery of nematocyst discharge. This likely indicates an interaction of compound **2** with the extracellular domain or inner pore of the mechanosensory ion channel as also suggested for STM (Fig. 3c)^22^. To obtain further evidence for possible direct binding of compound **2** to the cnidocil-associated TRPN channel, we performed an immunofluorescence staining of Hydra whole-mounts using the previously described pan-TRPN antibody^15^. Hydras were treated for 2 h with compound **2** or DMSO as a control and then fixed for immunostaining. As shown in Fig. 3d,e, the treatment led to a loss of the prominent signal at the base of the cnidocil, indicating relocalization of the TRPN channel protein within the battery cell. This could be a consequence of reduced TRPN interaction with other components of the mechanosensory apparatus as previously observed in *Xenopus* hair cells upon treatment with calcium chelators^23^.

**Figure 4:**
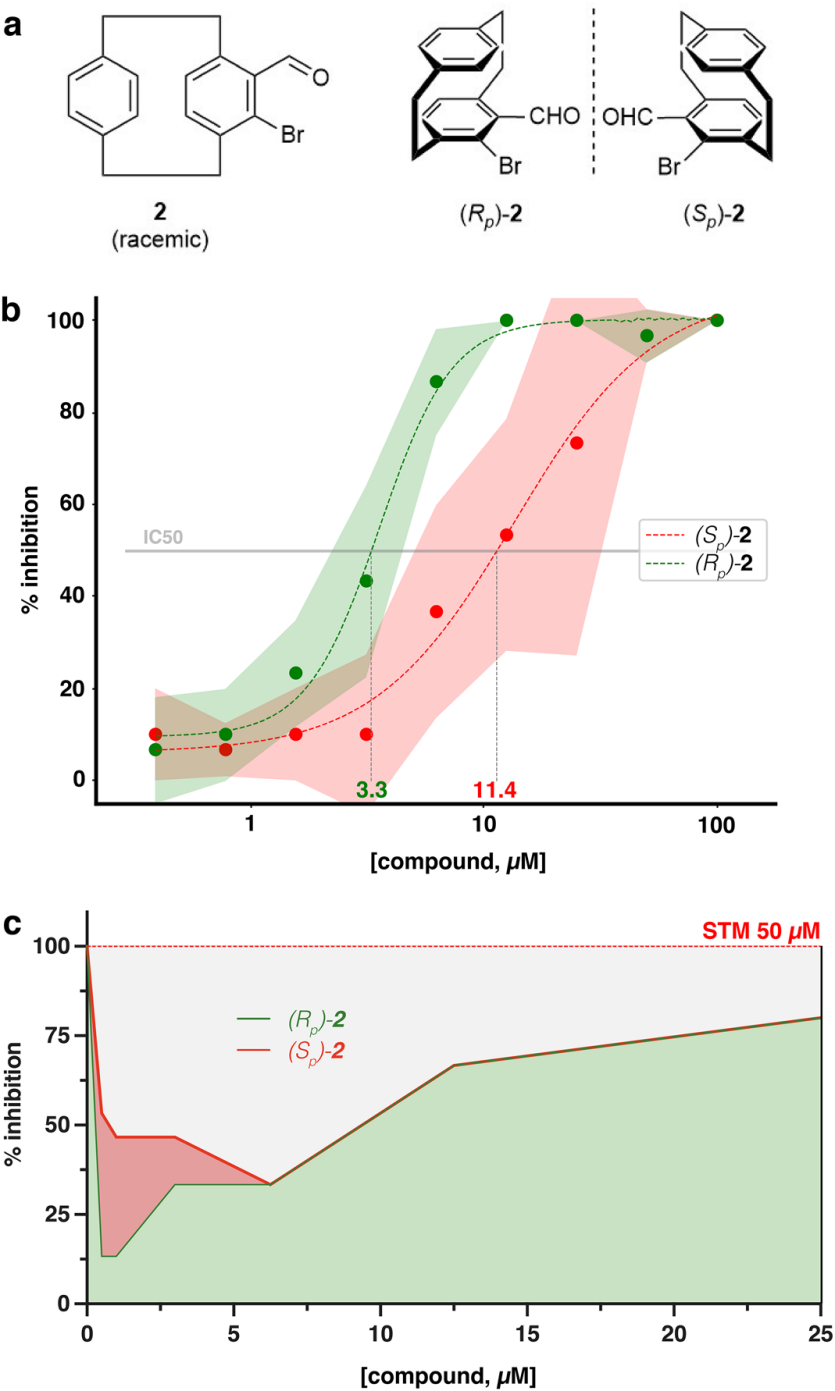
(**a**) Structures of racemic lead structure **2** and enantiomers (*S*_p_)-**2** and (*R*_p_)-**2**. (**b**) Dose–response study of the two enantiomers. Animals were treated in triplicates with the indicated concentration of the isomers for 30 min. Nematocyst discharge was recorded by counting the fraction of artemia (out of ten) caught by each polyp after 15 min. The compound concentration is shown in the log scale. Colored areas correspond to standard deviation. IC50 values were determined by fitting logistic functions to the average inhibition (dashed lines) and solving them for 50% inhibition. (**c**) Competition between compound **2** isomers and STM. Complete prey capture inhibition by 50 µM STM was partly neutralized by adding the enantiomers (*S*_p_)-**2** and (*R*_p_)-**2.** The neutralizing effect was more pronounced in the low micromolar concentration range and stronger for (*R*_p_)-**2.** Animals were treated in triplicates with the indicated concentration of the isomers in combination with 50 µM STM for 30 min. Prey capture was recorded as in the primary screen. STM by itself completely inhibited prey capture at 50 µM.

In order to investigate the contribution of the stereoisomers (*S*_*p*_)-**2** and (*R*_*p*_)-**2** (Fig. 4a) to the effect on nematocyst discharge that was observed for the racemic compound **2**, the respective compounds were synthesized and tested in a dose–response study (Fig. 4b). While at concentrations above 50 µM both isomers show full reduction of the prey capture activity after 30 min, the isomer (*R*_*p*_)-**2** showed an IC50 value (3.3 µM), which is about half as effective compared to the racemic compound (1.56 µM, Fig. 3b), while the isomer (*S*_*p*_)-**2** was significantly less active (IC50 11.4 µM). At higher concentrations, toxicity effects may also influence the result as both compounds showed the toxicity of grade 2–3 in concentrations of 25 µM and higher. We also tested whether the isomers of compound-**2** acted as competitors of STM as we assumed that they might bind to the same target protein. When animals were incubated with increasing doses of the isomers in combination with 50 µM STM, which by itself exerts full inhibition, prey capture was restored up to 80% in the presence of (*R*_*p*_)-**2** while (*S*_*p*_)-**2** was less effective (Fig. 4c). The neutralizing effect was most pronounced in the lower micromolar concentration range, indicating that the inhibitory effect of the compound-**2** isomers dominates at higher doses.

### Detailed structure–function analysis of [2.2]paracyclophanes

**Figure 5:**
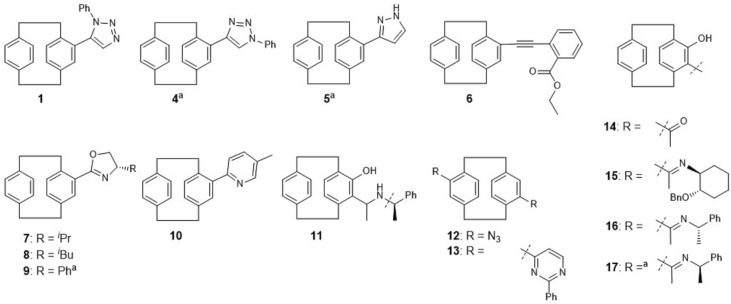
Selected [2.2]paracyclophanes with and without heterocyclic substitution. The origin and synthesis of the compounds are described in detail in Supplementary File 1 online. ^a^compound was not included in the primary assay.

The compound class of [2.2]paracyclophanes was found to be the most promising one for the identification of a possible inhibitor of nematocyst discharge and TRP modulation. During all the stages of the primary screen, [2.2]paracyclophanes showed the best results concerning their activity and toxicity. Therefore, further experiments were conducted with a selection of [2.2]paracyclophanes (Fig. 5), to be able to explore structure–activity relationships within this compound class in more detail (Table 1).

**Table 1:** Structure–activity relationships of [2.2]paracyclophane derivatives.

No	Compound	Rank^a^	Nematocyst discharge^b^		Toxicity^c^
			[50 µM]	[10 µM]	[10 µM]
1	**1**	1	0	0	N
2	**4**	–^d^	0	0	N
3	**5**	– ^d^	3	10	N
4	**6**	0	0	10	Y
5	**7**	2	0	0	Y
6	**8**	0	7	10	N
7	**9**	– ^d^	0	0	N
8	**10**	0	3	10	Y
9	**11**	0	0	0	N
10	**12**	3	10	10	N
11	**13**	0	10	10	N
12	**14**	1	0	10	N
13	**15**	0	0	7	N
14	**16**	1	0	0	N
15	**17**	– ^d^	10	10	N

^a^Indication after primary assay (10 µM): 0 = no prey capture in all animals tested (highly active) to 3 = prey capture in all animals as in control (no activity).

^b^Observations were recorded as means of biological triplicates using a well of 10 animals according to 0 = nematocyst discharge in no animal (high effect) to 10 = nematocyst discharge in all animals (no effect).

^c^Toxicity was recorded after 24 h at a concentration of 10 µM: Y = toxicity observed, N = no toxicity observed; toxicity included categories 2–5 (see figure legend Fig. 1).

^d^Was not included in the primary assay.

**Figure 6:**
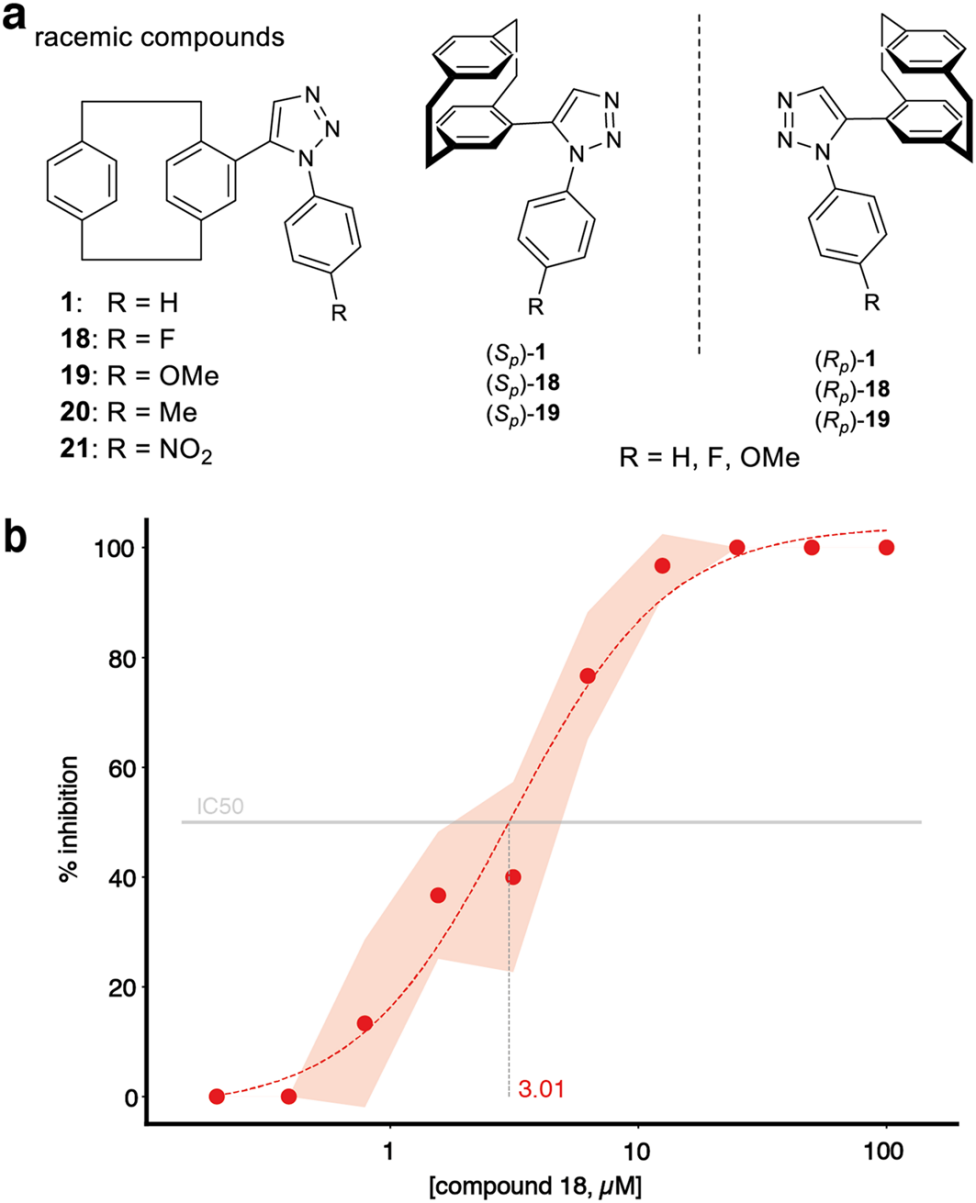
(**a**) Racemic lead structure **1** and structures of the 5-([2.2]paracyclophan-4-yl)-1-aryl-1,2,3-triazoles in their (*S*_*p*_)- and (*R*_*p*_)-configuration. The compounds were synthesized with different residues R: racemic compounds with R = H, F, OMe, Me, and NO_2_, *S*_p_ and *R*_p_-compounds with R = H, F and OMe each. Details on the structures and results can be retrieved from Supplementary File 1 online. (**b**) Dose–response study of racemic compound 18. Animals were treated in triplicates with the indicated concentration of the compound for 30 min. Nematocyst discharge was recorded by counting the fraction of artemia (out of ten) caught by each polyp after 15 min. The compound concentration is shown in the log scale. Colored areas correspond to standard deviation. IC50 values were determined by fitting logistic functions to the average inhibition (dashed lines) and solving them for 50% inhibition.

We aimed to confirm the potential of compound **1** in comparison to 14 other [2.2]paracyclophanes based on a prey capture assessment at concentrations of 50 µM and 10 µM. We could show that compound **1**, which belonged to the most active compounds in the primary assays (column “rank”, Table 1), was also one of the most potent inhibitors in this assay compared to a diverse set of other [2.2]paracyclophanes. Given that compound **1** gave highly reproducible good results in the dose–response studies and mechanosensory nematocyst discharge experiments (Supplementary Fig. S1 online, Fig. 4a, Table 1), we selected it as a lead structure for further analysis (Fig. 6). Our decision was also based on the fact that, in contrast to compounds **2** and **3**, the core of compound **1** offers manifold options to tune the structure and properties of the molecule by slight modifications.

According to known procedures in the literature^24–26^, a small set of 5-([2.2]paracyclophan-4-yl)-1-aryl-1,2,3-triazoles, the derivatives of which contained different substitutions in para-position of the phenyl group in the lead structure **1**, was synthesized (Fig. 6). Experiments were performed in a group of altogether 11 different 1,5-triazole-substituted [2.2]paracyclophanes consisting of racemic mixtures and enantiomerically pure compounds to investigate the effect of the single stereoisomers on the inhibitory effect of the [2.2]paracyclophanes on nematocyst discharge, as well as the toxicity of the compounds for the animals (Fig. 6). The evaluation of the compounds revealed that most of the chosen variations of the residues R (see Supplementary Table S2 online) are tolerated in terms of a strong nematocyst discharge inhibition. Four compounds (R = H, F, OMe, Me) showed a full discharge inhibition in concentrations of up to 5 µM, as a racemic mixture and also as enantiopure compounds in (*S*_p_)- and (*R*_p_)-configuration. Only the compound with R = NO_2_ (**21**) showed a lower effect. In summary, the best results were obtained with racemic compound **18** (R = F), for which we were able to show a significant effect on nematocyst discharge inhibition at a concentration of 0.5 µM and an IC50 value of 3.01 µM (Fig. 6b). While a general dependency of the observed activity on the stereoisomer of a chemical compound is expected for biological systems, we could not show such a dependency for the exposure of Hydras to (*S*_p_)- and (*R*_p_)-[2.2]paracyclophane derivatives (Supplementary Table S2 online).

## Discussion

In the present study, we carried out a small molecule screen to identify compounds that affect nematocyst discharge in *Hydra*. After a multi-stage screen, starting with 700 compounds randomly selected from different compound classes, we identified three compounds that showed complete inhibition of mechanosensory nematocyst discharge at low micromolar concentrations without apparent toxic effects. One of these compounds, a [2.2] paracyclophane was shown to inhibit nematocyst discharge reversibly and induce relocalization of the cnidocil-associated TRPN channel, indicating a possible direct interaction with a mechanosensory ion channel. We cannot exclude, though, that additional factors of the mechanosensory transduction machinery constituting the cnidocil apparatus might also be targeted. BLAST searches in the *Hydra* genome have identified cadherin-23 (XP_012559514.1) and calcium and integrin-binding family member 2 (CIB2) (XP_002155581.1) homologs that have been described as core components of the mechanosensory transduction machinery of mammalian inner ear hair cells ^27^.

Several other [2.2]paracyclophans were shown to inhibit nematocyst discharge in the low micromolar range; therefore the compound class was further examined to identify new inhibitory lead structures. Structure–activity analyses revealed common features that are required for the inhibitory activity of the [2.2]paracyclophane core motif. Triazoloparacyclophanes, which were identified as promising compounds providing a triazole ring as potent feature for further modifications, were used as lead structure to synthesize new derivatives. The first results on the investigation of these compounds give promising results with respect to the activity of triazoloparacyclophanes but also reveal toxicity of the latter compound class depending on the concentration and substituents. Our study demonstrates that *Hydra* can serve as a model for small molecule screens targeting mechanosensory ion channel activity in native tissues and describes the identification of [2.2]paracyclophanes as the promising compound class for designing selective inhibitors.

## Materials and methods

### Animals

*Hydra magnipapillata* strain 105 was used for all experiments. All animals were maintained in artificial *Hydra* medium (HM, 1 mM CaCl_2_, 0.1 mM MgCl_2_, 0.1 mM KCl, 1 mM NaH_2_CO_3_, pH 7.8) at 18 °C in polystyrene dishes (Carl Roth, Karlsruhe, Germany) and fed two to three times per week with freshly hatched *Artemia salina* nauplii. The medium was renewed 3–4 h after feeding and again the following day.

### Primary inhibitor screen

Similar sized animals were starved for 48 h prior to experiments, and 5 Hydras were transferred to each well of a 24-well plate in 1.5 ml HM. Compounds were solubilized in DMSO (10 mM) and added to the wells in triplicates at 10 µM final concentration. After 30 min incubation time at 18 °C, artemia were added to the wells and prey capture was evaluated after 15 min by visual inspection using light microscopy. The prey capture inhibition was recorded using a scale from 0 (full prey capture inhibition in all animals) to 3 (normal prey capture in all animals as in control). Intermediate grades of prey capture were given as 1 (prey capture in 1–2 animals with a markedly reduced number of caught artemia per animal) and 2 (prey capture in 3–4 animals with a slightly reduced number of caught artemia per animal). Full prey capture is defined as immediate immobilization of artemia by tentacle contact and attachment of several immobilized artemia to each tentacle. Streptomycin at 100 µM was used as positive control and HM with 0.001% DMSO as a negative control. Toxicity was evaluated after a period of 48 h in a parallel experiment without the addition of artemia and according to the classification described in the legend to Fig. 1.

### Dose-dependent prey capture inhibition assay

Similar sized animals were starved for 48 h prior to experiments, and one Hydra was transferred to the center of each well of a 48-well plate. Compounds were added to the wells in triplicates at given concentrations of a dilution series ranging from 0.2 to 50 µM and incubated for 30 min at 18 °C. Then 10 artemia were added to each well, and prey capture was evaluated after 15 min by counting the number of artemia attached to the tentacles using light microscopy. Animals tested positive in this assay were challenged by touching the tentacles shortly with the tip of a glass pipette to confirm the full mobility of the polyp. Hydras react to this stimulus by contraction of tentacles and body. In addition, toxicity was re-evaluated at this stage of the screen and compounds showing toxicity levels 3–5 (see legend to Fig. 1) were excluded. As a control, animals were incubated in HM containing DMSO at concentrations corresponding to the amounts added in the compound dilution series. IC50 values were determined by fitting logistic curves f(x) = L/(1 + e^(-k(x-x0))^) (L = max value of the curve, k = logistic growth rate/steepness, x_0_ = sigmoid midpoint) to the average dose-dependent inhibition response of the different chemicals using a custom python script (Supplementary File 3 online) and solving the resulting functions for inhibition at 50%.

### Assays for mechanosensory discharge inhibition

Similar sized animals were starved for 7 days prior to experiments, and 10 Hydras were transferred to each well of a 24-well plate. Compounds were added to the wells in triplicates at indicated concentrations. After 30 min of incubation, the tentacles of each animal were touched for 1–2 s with the tip of a clean glass pipette. Animals whose tentacles stayed attached to the glass pipette upon retraction were recorded as showing normal nematocyst discharge. For the reversibility assay, a short segment of a nylon fishing line was used as a probe according to Watson & Hessinger ^28^. The fishing line was coated at one end with 30% gelatin and after contacting tentacles, stenoteles having discharged into the gelatin were counted by phase-contrast microscopy. After assaying the inhibitory compounds at a common minimal concentration for full prey capture inhibition (12.5 µM), the medium was exchanged for HM, and animals were challenged again using fresh fishing lines.

### Immunocytochemistry

*Hydra magnipapillata* were relaxed in 2% urethane in HM and then fixed in freshly prepared ice-cold methanol for 4 h. Samples were rehydrated in 5-min steps using 75%, 50%, 25% ethanol in PBS, followed by washing steps: 3 X 0.1%/Tween-20/PBS, 3 X 0.1% Triton X100/PBS, 3 X 0.1%/ Tween-20/PBS, 10 min each. Samples were incubated in PBS with 1% BSA for 1 h before being incubated overnight at 4 °C in the same solution with the primary pan-TRPN antibody (1:200). To remove unbound antibodies, three washing steps with PBS were performed for 10 min each. The incubation with the secondary antibody (anti-rabbit, CF568) at 1:400 in PBS 1% BSA was performed for 2 h at room temperature. The animals were washed 3 times with PBS and then mounted on object slides with Mowiol. Images were acquired with a Nikon A1 confocal microscope using imaging software NIS Elements (AR 45.51.01, 3.10, SP3, Hotfix, Build645, https://www.gvsu.edu/cms4/asset/8FCAC028-902A-3EFC-5137403A360C8843/user_guide_nis-elements_ar.pdf). Further image processing was performed with Adobe Photoshop CS6 and Fiji.

### Chemical compounds

Detailed information on the origin and synthesis of the compounds used in this study are given in Supplementary File 1 online. Supplementary File 2 online provides a list of the compounds used in this study with identifiers of the compounds in the Molecule Archive (KIT-Karlsruhe). Several compounds that were used for this study were taken from the compound libraries of the Molecule Archive. The Molecule Archive collects synthesis products and archives them for re-use in future studies and for collaborations with other scientists. The labels of the molecules as they are registered in the compound platform are given in list form as additional information, which allows interested readers to obtain this material for comparison or further studies as long as the stock is available. Please refer to the given numbers in Supplemental File 2 online and contact the Molecule Archive (nicole.jung@kit.edu, anke.deckers@kit.edu, braese@kit.edu). To synthesize the molecules in the Molecule Archive, we refer to the published literature, the analysis of these molecules is added here to prove the identity of the compounds. For all compounds that were synthesized for the present study, the following chapter describes the syntheses and analysis. Additional information and the original data can be obtained from the repository Chemotion: www.chemotion-repository.net/home.

### NMR studies

^1^H-NMR spectra were recorded on a BRUKER Ascend 400 (400 MHz) and BRUKER AM 500 (500 MHz) spectrometer. Chemical shifts are given in parts per million (δ/ppm), downfield from tetramethylsilane (TMS) and are referenced to chloroform (7.27 ppm) as internal standard. All coupling constants are absolute values and J values are expressed in Hertz (Hz). The description of signals includes: s = singlet, br. s = broad singlet, d = doublet, t = triplet, dd = doublet of doublets, ddd = doublet of doublet of doublet, td = triplet of doublet, dt = doublet of triplets, q = quartet, quin = quintet. The spectra were analyzed according to the first order.^13^C NMR and ^13^C Gel-NMR spectra were recorded on Bruker Ascend 400 spectrometer. Chemical shifts are expressed in parts per million (δ/ppm) downfield from tetramethylsilane (TMS) and are referenced to chloroform (77.0 ppm) as internal standard. MS (EI) (electron impact mass spectrometry) and FAB (Fast Atom Bombardment) measurements were done with the instrument Finnigan MAT 95 (70 eV). For APCI spectra, the instrument expression CMS from Advion was used. The molecular fragments are quoted as the relationship between mass and charge (m/z), the intensities as a percentage value relative to the intensity of the base signal (100%). IR (infrared spectroscopy): ATR spectra were recorded by diamond crystal on Alpha IR. Routine monitoring of reactions were performed using silica gel coated aluminum plates (Merck, silica gel 60, F_254_) which were analyzed under UV-light at 254 nm and/or dipped into a solution of Seebach reagent (2.5% phosphor molybdic acid, 1.0% Cerium(IV) sulfate tetrahydrate and 6.0% sulfuric acid in H_2_O) and heated via hot air flow. Solvent mixtures are understood as v/v. Solvents, reagents and chemicals were purchased from Sigma Aldrich/Merck, Alfa Aesar, ABCR, and VWR and used without further purification unless stated otherwise.

## Supplementary Information

Supplementary Information 1.

Supplementary Information 2.

Supplementary Information 3.

## Data Availability

The datasets generated during and/or analyzed during the current study are available from the corresponding authors on reasonable request.
