# Supplementary material for: A small molecule screen identifies novel inhibitors of mechanosensory nematocyst discharge in Hydra
Source: Sci Rep. 2021 Oct 18;11:20627. doi: 10.1038/s41598-021-99974-7 (PMC8523708; doi:10.1038/s41598-021-99974-7)
Supplement: Supplementary file 1 — Supplementary Information 1. [file 41598_2021_99974_MOESM1_ESM.docx]

**Supplementary Information**

**A small molecule screen identifies novel inhibitors of mechanosensory nematocyst discharge in *Hydra***

Diana Hofmann, Niharika Garg, Simone Grässle, Sylvia Vanderheiden, Bruno Gideon Bergheim, Stefan Bräse, Nicole Jung and Suat Özbek

**Supplementary Fig. S1**


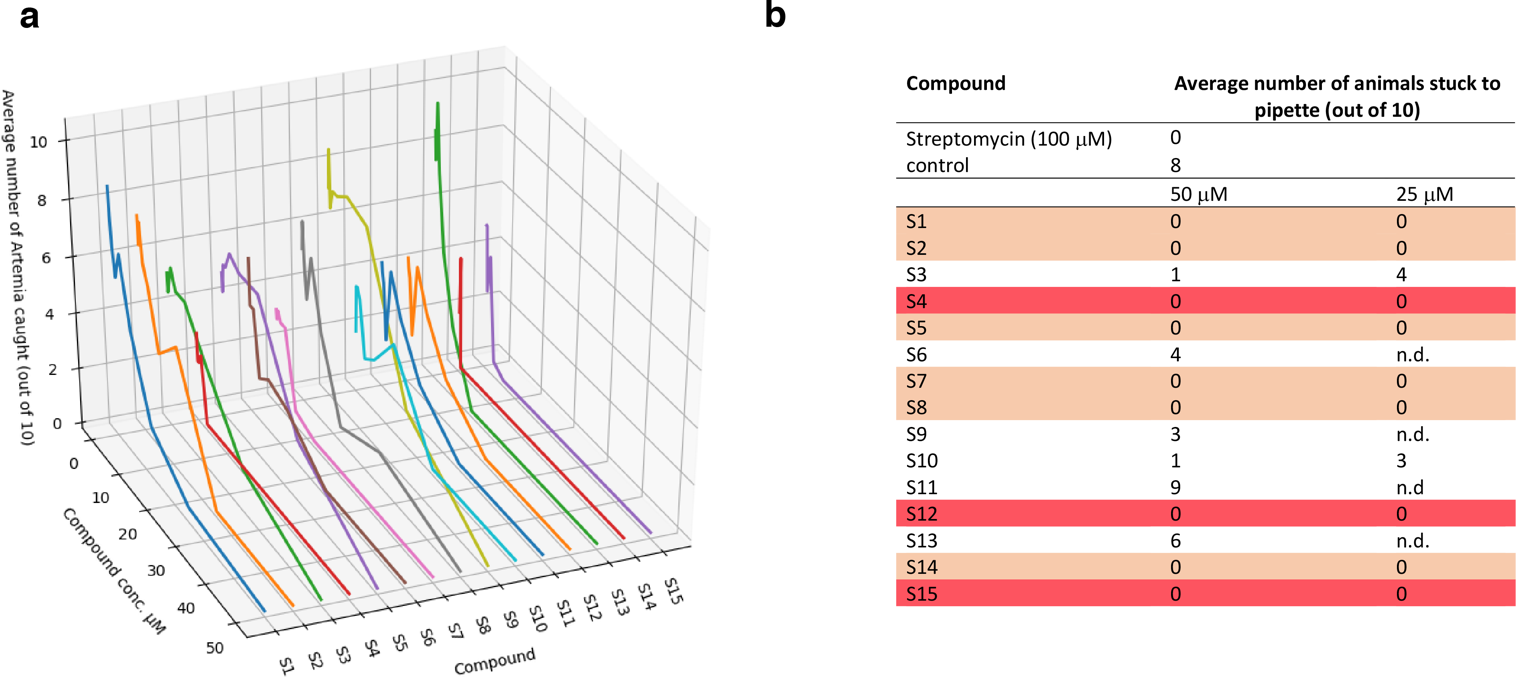


**Figure S1.** (a) Dose-response curves of 15 compounds (S1-S15) selected positive in second screening stage. The compounds were tested in triplicates for prey capture inhibition in a concentration range between 0.2-50 µM. Prey capture was evaluated by recording the average number of artemia (out of 10) caught by each polyp. The structures of the 15 selected compounds are given in the appendix below. (b) Evaluation of purely mechanical nematocyst discharge in starved animals. The candidate compounds were tested at 50 and 25 µM by recording the number of animals (out of ten) showing tentacle attachment to a glass pipette, indicating nematocyst discharge. Light red, full inhibition of nematocyst discharge at both concentrations applied. Dark red, compounds that in addition have shown a half-maximal inhibitory effect below 5 µM in (a). S4, S12 and S15 correspond to compounds 1, 2 and 3 (Fig. 3), respectively.

**Appendix to Figure S1.** Structures of 15 selected compounds tested in the dose-response studies.

**No canonical smiles**

**S1** C[C@@H](C(C)(C)C)/N=C(/c1c2ccc(c1O)CCc1ccc(CC2)cc1)\C

**S2:** O/N=C(/C(F)(F)F)\c1ccc(cc1)c1ccc(cc1)Br

**S3:** C/C(=N\[C@H](c1ccccc1)C)/c1c2ccc(c1O)CCc1ccc(CC2)cc1

**S4:** c1ccc(cc1)n1nncc1c1cc2ccc1CCc1ccc(CC2)cc1

**S5:** C/C(=N\[C@H]1CCCC[C@@H]1OCc1ccccc1)/c1c2ccc(c1O)CCc1ccc(CC2)cc1

**S6:** C#CCOB(n1c(C)cc(c1/C(=C/1\N=C(C=C1C)C)/c1c(F)c(F)c(c(c1F)F)F)C)F

**S7:** COC(=O)c1ccc(cc1)C1=C2C(=C(C(=N2[B](n2c1c(C)c(c2C)I)(F)F)C)I)C

**S8:** COC(=O)c1cccc(c1/N=N/N(C(C)C)C(C)C)C

**S9:** C1([2H])(SCCCS1)c1ccc(cc1)Br

**S10:** COC(=O)CC(=C1SCCCS1)/N=N/c1ccc(cc1)I

**S11:** OCc1cc(Br)ccc1Br

**S12:** O=Cc1c2CCc3ccc(CCc(c1Br)cc2)cc3

**S13:** CCCCOc1ccc(cc1)/N=N/c1ccc(cc1)CCCC

**S14:** C[N+](CCCCCCN(c1ccc(cc1)/C=C/c1ccc(cc1)/C=C/c1ccc(cc1)/C=C/c1ccc(cc1)N(CCCCCC[N+](C)(C)C)CCCCCC[N+](C)(C)C)CCCCCC[N+](C)(C)C)(C)C.I.[I-].[I-].[I-]

**S15:** O=C1C(=C(Br)C(=O)c2c1cccc2)Br

| L76 | L1 | L26 | L51 | L76 | L101 | L126 | L151 | L176 | L201 | L226 | L251 | L276 | L301 | L326 | L351 | L376 | L401 | L426 | L451 | L476 | L501 | L526 | L551 | L576 | L601 | L626 | L651 | L676 |
| --- | --- | --- | --- | --- | --- | --- | --- | --- | --- | --- | --- | --- | --- | --- | --- | --- | --- | --- | --- | --- | --- | --- | --- | --- | --- | --- | --- | --- |
| L77 | L2 | L27 | L52 | L77 | L102 | L127 | L152 | L177 | L202 | L227 | L252 | L277 | L302 | L327 | L352 | L377 | L402 | L427 | L452 | L477 | L502 | L527 | L552 | L577 | L602 | L627 | L652 | L677 |
| L78 | L3 | L28 | L53 | L78 | L103 | L128 | L153 | L178 | L203 | L228 | L253 | L278 | L303 | L328 | L353 | L378 | L403 | L428 | L453 | L478 | L503 | L528 | L553 | L578 | L603 | L628 | L653 | L678 |
| L79 | L4 | L29 | L54 | L79 | L104 | L129 | L154 | L179 | L204 | L229 | L254 | L279 | L304 | L329 | L354 | L379 | L404 | L429 | L454 | L479 | L504 | L529 | L554 | L579 | L604 | L629 | L654 | L679 |
| L80 | L5 | L30 | L55 | L80 | L105 | L130 | L155 | L180 | L205 | L230 | L255 | L280 | L305 | L330 | L355 | L380 | L405 | L430 | L455 | L480 | L505 | L530 | L555 | L580 | L605 | L630 | L655 | L680 |
| L81 | L6 | L31 | L56 | L81 | L106 | L131 | L156 | L181 | L206 | L231 | L256 | L281 | L306 | L331 | L356 | L381 | L406 | L431 | L456 | L481 | L506 | L531 | L556 | L581 | L606 | L631 | L656 | L681 |
| L82 | L7 | L32 | L57 | L82 | L107 | L132 | L157 | L182 | L207 | L232 | L257 | L282 | L307 | L332 | L357 | L382 | L407 | L432 | L457 | L482 | L507 | L532 | L557 | L582 | L607 | L632 | L657 | L682 |
| L83 | L8 | L33 | L58 | L83 | L108 | L133 | L158 | L183 | L208 | L233 | L258 | L283 | L308 | L333 | L358 | L383 | L408 | L433 | L458 | L483 | L508 | L533 | L558 | L583 | L608 | L633 | L658 | L683 |
| L84 | L9 | L34 | L59 | L84 | L109 | L134 | L159 | L184 | L209 | L234 | L259 | L284 | L309 | L334 | L359 | L384 | L409 | L434 | L459 | L484 | L509 | L534 | L559 | L584 | L609 | L634 | L659 | L684 |
| L85 | L10 | L35 | L60 | L85 | L110 | L135 | L160 | L185 | L210 | L235 | L260 | L285 | L310 | L335 | L360 | L385 | L410 | L435 | L460 | L485 | L510 | L535 | L560 | L585 | L610 | L635 | L660 | L685 |
| L86 | L11 | L36 | L61 | L86 | L111 | L136 | L161 | L186 | L211 | L236 | L261 | L286 | L311 | L336 | L361 | L386 | L411 | L436 | L461 | L486 | L511 | L536 | L561 | L586 | L611 | L636 | L661 | L686 |
| L87 | L12 | L37 | L62 | L87 | L112 | L137 | L162 | L187 | L212 | L237 | L262 | L287 | L312 | L337 | L362 | L387 | L412 | L437 | L462 | L487 | L512 | L537 | L562 | L587 | L612 | L637 | L662 | L687 |
| L88 | L13 | L38 | L63 | L88 | L113 | L138 | L163 | L188 | L213 | L238 | L263 | L288 | L313 | L338 | L363 | L388 | L413 | L438 | L463 | L488 | L513 | L538 | L563 | L588 | L613 | L638 | L663 | L688 |
| L89 | L14 | L39 | L64 | L89 | L114 | L139 | L164 | L189 | L214 | L239 | L264 | L289 | L314 | L339 | L364 | L389 | L414 | L439 | L464 | L489 | L514 | L539 | L564 | L589 | L614 | L639 | L664 | L689 |
| L90 | L15 | L40 | L65 | L90 | L115 | L140 | L165 | L190 | L215 | L240 | L265 | L290 | L315 | L340 | L365 | L390 | L415 | L440 | L465 | L490 | L515 | L540 | L565 | L590 | L615 | L640 | L665 | L690 |
| L91 | L16 | L41 | L66 | L91 | L116 | L141 | L166 | L191 | L216 | L241 | L266 | L291 | L316 | L341 | L366 | L391 | L416 | L441 | L466 | L491 | L516 | L541 | L566 | L591 | L616 | L641 | L666 | L691 |
| L92 | L17 | L42 | L67 | L92 | L117 | L142 | L167 | L192 | L217 | L242 | L267 | L292 | L317 | L342 | L367 | L392 | L417 | L442 | L467 | L492 | L517 | L542 | L567 | L592 | L617 | L642 | L667 | L692 |
| L93 | L18 | L43 | L68 | L93 | L118 | L143 | L168 | L193 | L218 | L243 | L268 | L293 | L318 | L343 | L368 | L393 | L418 | L443 | L468 | L493 | L518 | L543 | L568 | L593 | L618 | L643 | L668 | L693 |
| L94 | L19 | L44 | L69 | L94 | L119 | L144 | L169 | L194 | L219 | L244 | L269 | L294 | L319 | L344 | L369 | L394 | L419 | L444 | L469 | L494 | L519 | L544 | L569 | L594 | L619 | L644 | L669 | L694 |
| L95 | L20 | L45 | L70 | L95 | L120 | L145 | L170 | L195 | L220 | L245 | L270 | L295 | L320 | L345 | L370 | L395 | L420 | L445 | L470 | L495 | L520 | L545 | L570 | L595 | L620 | L645 | L670 | L695 |
| L96 | L21 | L46 | L71 | L96 | L121 | L146 | L171 | L196 | L221 | L246 | L271 | L296 | L321 | L346 | L371 | L396 | L421 | L446 | L471 | L496 | L521 | L546 | L571 | L596 | L621 | L646 | L671 | L696 |
| L97 | L22 | L47 | L72 | L97 | L122 | L147 | L172 | L197 | L222 | L247 | L272 | L297 | L322 | L347 | L372 | L397 | L422 | L447 | L472 | L497 | L522 | L547 | L572 | L597 | L622 | L647 | L672 | L697 |
| L98 | L23 | L48 | L73 | L98 | L123 | L148 | L173 | L198 | L223 | L248 | L273 | L298 | L323 | L348 | L373 | L398 | L423 | L448 | L473 | L498 | L523 | L548 | L573 | L598 | L623 | L648 | L673 | L698 |
| L99 | L24 | L49 | L74 | L99 | L124 | L149 | L174 | L199 | L224 | L249 | L274 | L299 | L324 | L349 | L374 | L399 | L424 | L449 | L474 | L499 | L524 | L549 | L574 | L599 | L624 | L649 | L674 | L699 |
| L100 | L25 | L50 | L75 | L100 | L125 | L150 | L175 | L200 | L225 | L250 | L275 | L300 | L325 | L350 | L375 | L400 | L425 | L450 | L475 | L500 | L525 | L550 | L575 | L600 | L625 | L650 | L675 | L700 |
|  | **1** |  | **2** |  | **3** | | |  | **4** | |  | **5** |  | **6** | |  |  |  | **7** |  | **8** |  |  | **9** | |  |  | **10** |

**Supplementary Table S1**. Primary assay with 700 compounds (L1-L700) detailing the heat map given in Figure 2a. Boxed areas indicate compound classes as follows. 1: Pyrazoles, 2: Triazenes, 3: Paracyclophanes, 4: Coumarins, 5: Sugars, 6: Fluorinated compound, 7: Piperazines, 8: Steroids, 9: Azo-Containing compounds, 10: Indoles, not boxed: compounds with no assignment (NA). Colors indicate effective nematocyst discharge derived from prey capture efficiency. Grey, grade 3 (prey capture as in control); yellow, grade 2; orange, grade 1; red, grade 0 (full prey capture inhibition).

**Supplementary Table S2**. Detailed investigation of nematocyst discharge inhibition depending on the substitution pattern of 5-([2.2]paracyclophan-4-yl)-1-aryl-1,2,3-triazoles and their configuration. Referring to Figure 6 of the manuscript.

| **No** | **Cpd** | **R** | **Isomer-type** | **10 µM^a^** | **5 µM^a^** | **0.5 µM^a^** | **Tox^b^**  **[10 µM]** |
| --- | --- | --- | --- | --- | --- | --- | --- |
| 1 | **1** | H | rac-**1** | 1 | 0 | 10 | - |
| 2 |  | H | (*S*_p_)-**1** | 0 | 0 | 10 | - |
| 3 |  | H | (*R*_p_)-**1** | 0 | 0 | 9 | Y |
| 4 | **18** | F | Rac-**18** | 0 | 0 | 8 | - |
| 5 |  | F | (*S*_p_)-**18** | 0 | 0 | 6 | - |
| 6 |  | F | (*R*_p_)-**18** | 0 | 0 | 8 | Y |
| 7 | **19** | OMe | Rac-**19** | 0 | 0 | 10 | - |
| 8 |  | OMe | (*S*_p_)-**19** | 0 | 1 | 10 | - |
| 9 |  | OMe | (*R*_p_)-**19** | 0 | 0 | 10 | - |
| 10 | **20** | Me | rac-**20** | 0 | 0 | 10 | - |
| 11 | **21** | NO_2_ | rac-**21** | 2 | 10 | 10 | - |

^a^Observations were recorded as means of biological triplicates using a well of 10 animals according to 0 = nematocyst discharge in no animal (high effect) to 10 = nematocyst discharge in all animals (no effect), ^b^ Toxicity was recorded after 24 h at 10 µM, Y = toxicity observed; - = no or low toxicity observed.

# Supplementary File 1. Origin and synthesis of compounds used in this study.

**Origin and synthesis of the precursors for compounds described in Figures 4 and 6.**

**Supplementary Scheme 1.** Synthesis of the Precursors (*S*_P_)-**S19** and (*R*_P_)-**S19** according to literature known protocols. ^[25]^

**(*S*_P_,*R*)-(1-(1,4(1,4)-Dibenzenacyclohexaphane-1^2^-yl)-*N*-(1-phenylethyl)methanimine ((*S*_P_)-S18)**

The compound (*S*_P_)-**S18** was used as available in the Molecule Archive. The original syntheses can be retrieved from the literature. ^[25]^

**(*R*_P_,*S*)-1-(1,4(1,4)-Dibenzenacyclohexaphane-1^2^-yl)-*N*-(1-phenyl-2-(p-tolyl)ethyl)methanimine ((*R*_P_)-S20)**

The compound (*R*_P_)-**S20** was taken as available in the Molecule Archive. The original syntheses can be retrieved from the literature ^[27]^.

**(*R*_P_)-1,4(1,4)-dibenzenacyclohexaphane-1^2^-carbaldehyde ((*R*_P_)-S19)**


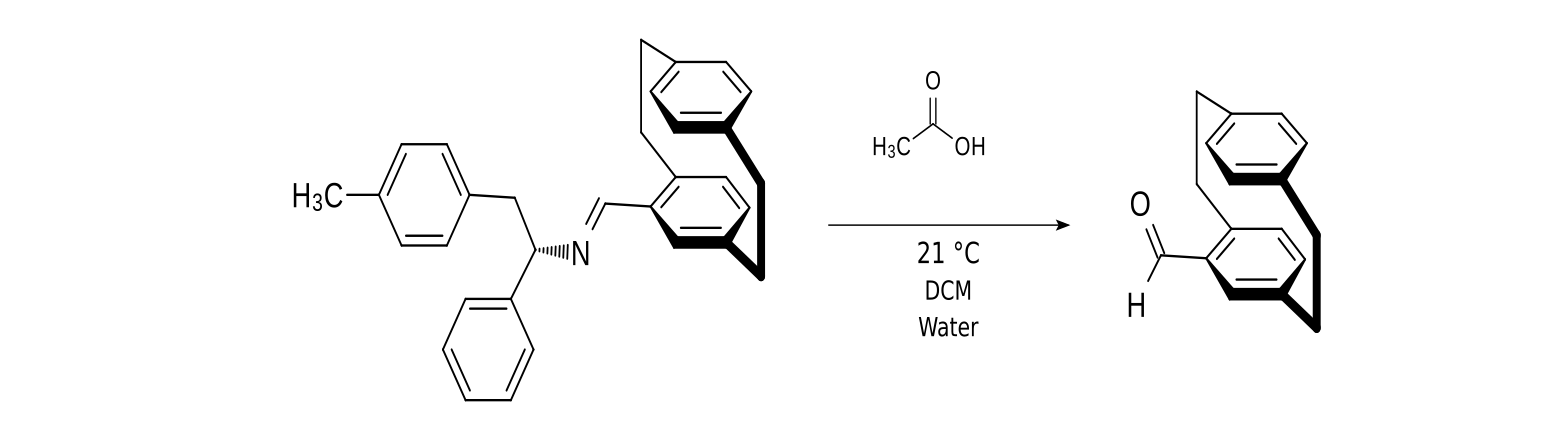


Smiles: O=Cc1cc2CCc3ccc(CCc1cc2)cc3; InChIKey: BAIBHOHKSYVVCM-UHFFFAOYSA-N

The starting material (*R*_P_,*S*)-1-(1,4(1,4)-dibenzenacyclohexaphane-1^2^-yl)-*N*-(1-phenyl-2-(p-tolyl)ethyl)methanimine (1.33 g, 3.09 mmol, 1.00 equiv) was dissolved in methylene chloride (55.0 mL) and dist. water (750 μL). Acetic acid (0.788 g, 13.1 mmol, 4.24 equiv) was added and the reaction mixture was stirred for 16 hours at 21 °C. The layers were separated and the organic phases were washed with 30 mL of 5% HCl solution and water until it was colorless. The organic phase was dried over Na_2_SO_4_, filtered and the solvent was evaporated under reduced pressure. The crude product was purified by flash chromatography (Interchim, Puriflash, 100 g silica gel) in cyclohexane:ethyl acetate (gradient: 1:0 to 10:1) and the target compound was obtained in 96% yield (701 mg, 2.97 mmol).

*R_f_* = 0.37 (cyclohexane/ethyl acetate 10:1). ^1^H NMR (400 MHz, CDCl_3_, ppm) δ = 9.95 (s, 1H), 7.02–7.01 (m, 1H), 6.77–6.69 (m, 1H), 6.63–6.32 (m, 5H), 4.11 (ddd, *J* = 13.0 Hz, *J* = 9.9 Hz, *J* = 1.8 Hz, 1H), 3.26 (ddd, *J* = 14.8 Hz, *J* = 11.4 Hz, *J* = 2.7 Hz, 1H), 3.22–3.00 (m, 5H), 2.95 (ddd, *J* = 13.1 Hz, *J* = 10.1 Hz, *J* = 6.7 Hz, 1H). Spectrum contains residues of H_2_O; ^13^C NMR (100 MHz, CDCl_3_ ppm) δ = 192.1, 143.4, 140.8, 139.7, 139.6, 138.2, 136.8, 136.5, 136.3, 133.4, 133.1, 132.5, 132.3, 35.4, 35.3, 35.2, 33.8; APCI-MS (*m*/*z*): 430 (26), 326 (32), 237 (100) [M+1]^+^, 163 (17). HRMS (C_17_H_16_O): Calcd 236.1201, Found 236.1190; IR (ATR, ṽ) = 3033, 3012, 2956, 2922, 2890, 2850, 2758, 1676, 1589, 1554, 1496, 1489, 1448, 1436, 1411, 1285, 1227, 1207, 1180, 1160, 1145, 1116, 1102, 1006, 976, 963, 936, 909, 874, 796, 773, 742, 718, 701, 657, 636, 622, 577, 572, 517, 497, 462, 452, 433, 390 cm^–1^.

Additional information on the chemical synthesis is available via Chemotion repository:

<https://dx.doi.org/10.14272/reaction/SA-FUHFF-UHFFFADPSC-BAIBHOHKSY-UHFFFADPSC-NUHFF-NUHFF-NUHFF-ZZZ.1>

Additional information on the analysis of the target compound is available via Chemotion repository:

<https://dx.doi.org/10.14272/BAIBHOHKSYVVCM-UHFFFAOYSA-N.2>

**(*S*_P_)-1,4(1,4)-Dibenzenacyclohexaphane-1^2^-carbaldehyde ((*S*_P_)-S19)**

Smiles: O=Cc1cc2CCc3ccc(CCc1cc2)cc3; InChIKey: BAIBHOHKSYVVCM-UHFFFAOYSA-N

(*S*_P_)-1-(1,4(1,4)-Dibenzenacyclohexaphane-1^2^-yl)-*N*-(1-phenylethyl)methanimine (1.42 g, 4.2 mmol, 1.00 equiv) was hydrolyzed by column chromatography on silica gel with methylene chloride. The target compound was obtained in quantitative yield (1.03 g, 4.37 mmol).

*R_f_* = 0.57 (cyclohexane/ethyl acetate 4:1). ^1^H NMR (400 MHz, CDCl3, ppm) δ = 9.95 (s, 1H), 7.01 (d, *J* = 1.9 Hz, 1H), 6.76–6.68 (m, 1H), 6.62–6.54 (m, 2H), 6.53–6.46 (m, 1H), 6.45–6.35 (m, 2H), 4.11 (ddd, *J* = 13.1 Hz, *J* = 9.8 Hz, *J* = 1.8 Hz, 1H), 3.31 – 3.00 (m, 6H), 2.95 (ddd, *J* = 13.1 Hz, *J* = 10.2 Hz, *J* = 6.7 Hz, 1H) ; ^13^C NMR (100 MHz, CDCl3, ppm) δ = 192.1, 143.4, 140.8, 139.7, 139.6, 138.2, 136.8, 136.5, 136.3, 133.4, 133.1, 132.5, 132.3, 35.4, 35.3, 35.1, 33.8; EI (m/z, 70 eV, 50 °C): 236 (54), 131 (14), 104 (100), 78 (12). HRMS (C_17_H_16_O): Calcd 236.1201, Found 236.1200; IR (ATR, ṽ) = 3033, 3012, 2956, 2922, 2890, 2850, 2758, 1674, 1588, 1553, 1496, 1489, 1436, 1411, 1395, 1323, 1285, 1227, 1205, 1180, 1160, 1145, 1116, 1102, 965, 935, 908, 874, 816, 796, 773, 742, 718, 701, 657, 636, 620, 578, 572, 517, 497, 465, 452, 433, 424, 385 cm^–1^.

Additional information on the chemical synthesis is available via Chemotion repository:

<https://dx.doi.org/10.14272/reaction/SA-FUHFF-UHFFFADPSC-BAIBHOHKSY-UHFFFADPSC-NUHFF-NUHFF-NUHFF-ZZZ.2>

Additional information on the analysis of the target compound is available via Chemotion repository:

<https://dx.doi.org/10.14272/BAIBHOHKSYVVCM-UHFFFAOYSA-N.3>

**2,3-Dibromonaphthalene-1,4-dione (3)**

Compound **3** was used as available in the Molecule Archive. The compound was synthesized by Irina Protasova during her PhD thesis in the Stefan Bräse Group, Karlsruhe. The original syntheses can be retrieved from the literature ^[27]^.

The reaction description and additional analytical information including original data files is available via Chemotion Repository: <https://dx.doi.org/10.14272/PSMABVOYZJWFBV-UHFFFAOYSA-N.1>

**Origin and Synthesis of compounds described in Fig. 4 and 6.**

**Supplementary Scheme 2.** Strategy to the synthesis of the [2.2]paracyclophanes **2**. *****only the structures of the synthesis of (*R*_P_)-**2** from (*S*_P_)-**S19** are shown. The synthesis of (*S*_P_)-**2** was done in the same manner.

**(*rac*)**-**1^3^-Bromo-1,4(1,4)-dibenzenacyclohexaphane-1^2^-carbaldehyde ((*rac*)-2)**

The compound (*rac*)-**2** was taken as available in the Molecule Archive. The original syntheses can be retrieved from the literature ^[28]^.

**(*R*_P_)-1,4(1,4)-Dibenzenacyclohexaphane-1^2^-carbaldehyde *O*-methyl oxime ((*R*_P_)-S25)**

Smiles: CO/N=C/c1cc2ccc1CCc1ccc(CC2)cc1; InChIKey: JKQHPBPRTZLGQK-CPNJWEJPSA-N

*O*-Methylhydroxylamine hydrochloride (127 mg, 1.5 mmol, 1.20 equiv) was in dry methylene chloride (7.0 mL) and pyridine (402 mg, 411 μL, 5.1 mmol, 4.00 equiv) dissolved under nitrogen-atmosphere and (*R*_P_)-1,4(1,4)-dibenzenacyclohexaphane-1^2^-carbaldehyde (300 mg, 1.3 mmol, 1.00 equiv) was added to the reaction. The reaction was stirred for 16 hours at room temperature. The crude product was purified by flash chromatography (Interchim, Puriflash, 25 g silica gel) in cyclohexane:ethyl acetate (gradient: 1:0 to 20:1) giving 294 mg (1.11 mmol) of the target compound in 87% yield.

*R_f_* = 0.53 (cyclohexane/ethyl acetate 10:1).^1^H NMR (400 MHz, CDCl_3_, ppm) δ = 8.09 (s, 1H), 6.79–6.78 (m, 1H), 6.67–6.44 (m, 6H), 4.04 (s, 3H), 3.62 (ddd, *J* = 13.5 Hz, *J* = 9.9 Hz, *J* = 1.8 Hz, 1H), 3.22–2.94 (m, 6H), 2.87 (ddd, *J* = 13.5 Hz, *J* = 10.3 Hz, *J* = 6.7 Hz, 1H). ^13^C NMR (100 MHz, CDCl_3_, ppm) δ = 148.1, 140.3, 139.5, 139.4, 139.3, 135.5, 134.0, 133.3, 133.0, 132.3, 132.1 (2C), 131.0, 62.1, 35.5, 35.2, 34.0, 34.1; APCI-MS (*m*/*z*): 266 (100) [M+1]^+^, 163 (74), 147 (24), 121 (16). HRMS (C_18_H_19_NO): Calcd 265.1467, Found 265.1454; IR (ATR, ṽ) = 2987, 2952, 2925, 2890, 2849, 2812, 1902, 1589, 1554, 1499, 1465, 1449, 1435, 1424, 1411, 1353, 1346, 1320, 1286, 1228, 1204, 1183, 1157, 1150, 1118, 1095, 1052, 973, 955, 938, 904, 873, 832, 816, 798, 764, 718, 696, 652, 643, 635, 584, 547, 528, 514, 490, 463, 399 cm^–1^.

Additional information on the chemical synthesis is available via Chemotion repository:

<https://dx.doi.org/10.14272/reaction/SA-FUHFF-UHFFFADPSC-JKQHPBPRTZ-UHFFFADPSC-NUHFF-NGROS-NUHFF-ZZZ>

Additional information on the analysis of the target compound is available via Chemotion repository:

<https://dx.doi.org/10.14272/JKQHPBPRTZLGQK-CPNJWEJPSA-N.1>

**(*S*_P_)-1^3^-Bromo-1,4(1,4)-dibenzenacyclohexaphane-1^2^-carbaldehyde *O*-methyl oxime** **((*S*_P_)-S26)**

Smiles: CO/N=C/c1c2ccc(c1Br)CCc1ccc(CC2)cc1; InChIKey: VQEGCJLHLBTWRF-UDWIEESQSA-N

(*R*_P_)-1,4(1,4)-Dibenzenacyclohexaphane-1^2^-carbaldehyde *O*-methyl oxime (232 mg, 875 μmol, 1.00 equiv) was added to a solution of 1-bromopyrrolidine-2,5-dione (187 mg, 1.1 mmol, 1.20 equiv), silver trifluoroacetate (38.7 mg, 175 μmol, 0.200 equiv) and palladium(II) acetate (39.3 mg, 175 μmol, 0.200 equiv) in dry 1,2-dichloroethane (17.00 mL) under nitrogen. The reaction was stirred for 3 hours at 100 °C. As preparation for the column chromatography (dryload), Celite was added (0.8 g) and the solvent was evaporated. The obtained crude product was purified via flash-chromatography (Interchim, Puriflash, 50 g silica gel) using cyclohexane/ethyl acetate (gradient: 1:0 to 20:1) giving 200 mg (580 µmol) of the target compound in 66% yield.

*R_f_* = 0.39 (cyclohexane/ethyl acetate 20:1). ^1^H NMR (400 MHz, CDCl_3_, ppm) δ = 8.17 (s, 1H), 7.02–6.93 (m, 1H), 6.65–6.46 (m, 5H), 4.05 (s, 3H), 3.92–3.84 (m, 1H), 3.50 (ddd, *J* = 13.1 Hz, *J* = 10.1 Hz, *J* = 2.5 Hz, 1H), 3.21–3.02 (m, 4H), 2.93–2.79 (m, 2H). The spectrum contains residues of cyclohexane and ca. 10% unknown impurities; ^13^C NMR (100 MHz, CDCl_3_, ppm) δ = 149.8, 141.6, 139.9, 139.6, 139.0, 134.9, 134.1, 133.4, 133.0, 131.7, 130.1, 129.5, 128.4, 62.3, 36.0, 35.0, 34.5, 33.3 The spectrum contains residues of cyclohexane; EI (m/z, 70 eV, 50 °C): 343/345 (12/13), 314/316 (21/18), 265 (31), 238/240 (13/13), 208/210 (18/22), 160 (30), 130 (52), 103/105 (27/27), 104 (100), 78 (14). HRMS (C_18_H_18_ONBr): Calcd 343.0572, Found 343.0571; IR (ATR, ṽ) = 3063, 3029, 3002, 2985, 2921, 2891, 2849, 2813, 1894, 1686, 1592, 1572, 1541, 1497, 1446, 1438, 1424, 1409, 1392, 1333, 1316, 1239, 1200, 1180, 1166, 1119, 1095, 1048, 982, 938, 915, 899, 891, 877, 837, 799, 769, 742, 717, 671, 646, 582, 564, 545, 516, 493, 469, 433, 405, 385, 378 cm^–1^.

Additional information on the chemical synthesis is available via Chemotion repository:

<https://dx.doi.org/10.14272/reaction/SA-FUHFF-UHFFFADPSC-VQEGCJLHLB-UHFFFADPSC-NUHFF-NXICJ-NUHFF-ZZZ.1>

Additional information on the analysis of the target compound is available via Chemotion repository:

<https://dx.doi.org/10.14272/VQEGCJLHLBTWRF-UDWIEESQSA-N.2>

**(*S*_P_)-1^3^-Bromo-1,4(1,4)-dibenzenacyclohexaphane-1^2^-carbaldehyde** **((*S*_P_)-2)**

Smiles: O=Cc1c2CCc3ccc(CCc(c1Br)cc2)cc3; InChIKey: HOTMMJRJOHGLRS-UHFFFAOYSA-N

(*S*_P_)-1^3^-Bromo-1,4(1,4)-dibenzenacyclohexaphane-1^2^-carbaldehyde *O*-methyl oxime (148 mg, 430 μmol, 1.00 equiv) was dissolved in THF (6.00 mL) and water (0.6 mL). 4-Toluenesulfonic acid monohydrate (163 mg, 163 μL, 859 μmol, 2.00 equiv) and formaldehyde (339 mg, 311 μL, 4.3 mmol, 10.0 equiv) were added. The reaction was stirred in a microwave for 4 hours at 120 °C, 10 bar and 30 Watt. The crude product was purified by flash chromatography (Interchim, Puriflash, 25 g silica gel) in cyclohexane:ethyl acetate (gradient: 1:0 to 10:1) giving 92.3 mg (0.293 mmol) of the target compound in 68% yield.

*R_f_* = 0.41 (cyclohexane/ethyl acetate 20:1). ^1^H NMR (400 MHz, CDCl_3_, ppm) δ = 10.09 (s, 1H), 7.00–6.92 (m, 1H), 6.70–6.56 (m, 3H), 6.52–6.44 (m, 1H), 6.43–6.35 (m, 1H), 3.92 (ddd, *J* = 12.5 Hz, *J* = 9.1 Hz, *J* = 3.1 Hz, 1H), 3.58 (ddd, *J* = 13.2 Hz, *J* = 10.3 Hz, *J* = 2.6 Hz, 1H), 3.26–3.10 (m, 3H), 3.08 (ddd, *J* = 13.2 Hz, *J* = 10.6 Hz, *J* = 2.6 Hz, 1H), 2.86 (dddd, *J* = 26.7 Hz, *J* = 12.8 Hz, *J* = 10.1 Hz, *J* = 6.2 Hz, 2H). ^13^C NMR (100 MHz, CDCl_3_, ppm) δ = 194.2, 144.9, 140.8, 139.8, 139.1, 138.6, 134.5, 134.2, 133.5, 132.9, 132.1, 131.2, 128.7, 35.3, 34.7, 34.5, 33.3; EI (m/z, 70 eV, 50 °C): 314/316 (25/25), 235 (11), 104 (100), 78 (10). HRMS (C_17_H_15_OBr): Calcd 314.0306, Found 314.0307; IR (ATR, ṽ) = 3007, 2935, 2921, 2891, 2847, 2796, 2765, 1900, 1686, 1594, 1571, 1533, 1497, 1455, 1434, 1407, 1368, 1320, 1259, 1244, 1225, 1193, 1174, 1163, 1118, 1095, 1054, 993, 972, 949, 943, 932, 892, 871, 839, 799, 769, 732, 714, 666, 653, 578, 538, 514, 490, 476, 448, 397 cm^–1^.

Additional information on the chemical synthesis is available via Chemotion repository:

<https://dx.doi.org/10.14272/reaction/SA-FUHFF-UHFFFADPSC-HOTMMJRJOH-UHFFFADPSC-NUHFF-NUHFF-NUHFF-ZZZ>

Additional information on the analysis of the target compound is available via Chemotion repository:

<https://dx.doi.org/10.14272/HOTMMJRJOHGLRS-UHFFFAOYSA-N.1>

**(*S*_P_)-1,4(1,4)-Dibenzenacyclohexaphane-1^2^-carbaldehyde *O*-methyl oxime ((*S*_P_)-S25)**

Smiles: CO/N=C/c1cc2ccc1CCc1ccc(CC2)cc1; InChIKey: JKQHPBPRTZLGQK-CPNJWEJPSA-N

*O*-Methylhydroxylamine hydrochloride (127 mg, 1.5 mmol, 1.20 equiv) was dissolved in dry methylene chloride (7.0 mL) and pyridine (402 mg, 411 μL, 5.1 mmol, 4.00 equiv) under nitrogen atmosphere and (*S*_P_)-1,4(1,4)-dibenzenacyclohexaphane-1^2^-carbaldehyde (300 mg, 1.3 mmol, 1.00 equiv) was added to the reaction. The reaction was stirred for 16 hours at room temperature. The crude product was purified by flash chromatography (Interchim, Puriflash, 25 g silica gel) in cyclohexane:ethyl acetate (gradient: 1:0 to 10:1) to give 321 mg (1.21 mmol) of the target compound in 95% yield.

*R_f_* = 0.48 (cyclohexane/ethyl acetate 10:1). ^1^H NMR (400 MHz, CDCl_3_, ppm) δ = 8.10 (s, 1H), 6.80–6.79 (m, 1H), 6.67–6.44 (m, 6H), 4.05 (s, 3H), 3.63 (ddd, *J* = 13.5 Hz, *J* = 9.9 Hz, *J* = 1.9 Hz, 1H), 3.23–2.94 (m, 6H), 2.87 (ddd, *J* = 13.4 Hz, *J* = 10.3 Hz, *J* = 6.7 Hz, 1H). ^13^C NMR (100 MHz, CDCl_3_, ppm) δ = 148.0, 140.3, 139.5, 139.4, 139.3, 135.4, 134.0, 133.2, 133.0, 132.3, 132.1 (2C), 131.0, 62.1, 35.5, 35.2, 35.0, 34.1; EI (m/z, 70 eV, 30 °C): 265 (23), 160 (52), 130 (100), 104 (51), 77 (24). HRMS (C_18_H_19_ON): Calcd 265.1467, Found 265.1467; IR (ATR, ṽ) = 2990, 2952, 2927, 2891, 2850, 2812, 1592, 1554, 1499, 1449, 1435, 1411, 1346, 1320, 1286, 1231, 1205, 1184, 1159, 1116, 1091, 1052, 972, 955, 939, 904, 874, 798, 764, 718, 696, 652, 643, 635, 584, 547, 528, 514, 492, 465, 398, 382 cm^–1^.

Additional information on the chemical synthesis is available via Chemotion repository:

<https://dx.doi.org/10.14272/reaction/SA-FUHFF-UHFFFADPSC-JKQHPBPRTZ-UHFFFADPSC-NUHFF-NGROS-NUHFF-ZZZ.1>

Additional information on the analysis of the target compound is available via Chemotion repository:

<https://dx.doi.org/10.14272/JKQHPBPRTZLGQK-CPNJWEJPSA-N.2>

**(*R*_P_)-1^3^-Bromo-1,4(1,4)-dibenzenacyclohexaphane-1^2^-carbaldehyde *O*-methyl oxime ((*R*_P_)-S26)**

Smiles: CO/N=C/c1c2ccc(c1Br)CCc1ccc(CC2)cc1; InChIKey: VQEGCJLHLBTWRF-UDWIEESQSA-N

The starting material (*S*_P_)-1,4(1,4)-dibenzenacyclohexaphane-1^2^-carbaldehyde *O*-methyl oxime (100 mg, 377 μmol, 1.00 equiv) was added to a solution of 1-bromopyrrolidine-2,5-dione (80.5 mg, 452 μmol, 1.20 equiv), silver trifluoroacetate (16.6 mg, 75 μmol, 0.200 equiv) and palladium(II) acetate (16.9 mg, 75 μmol, 0.200 equiv) in dry 1,2-dichloroethane (7.00 mL) under nitrogen and the reaction was stirred for 3 hours at 100 °C. The crude product was purified by flash chromatography (Interchim, Puriflash, 25 g silica gel) in cyclohexane:ethyl acetate (gradient: 1:0 to 10:1) and 59.4 mg (0.173 mmol) of the target compound was obtained in 46% yield.

*R_f_* = 0.39 (cyclohexane/ethyl acetate 20:1). ^1^H NMR (400 MHz, CDCl_3_, ppm) δ = 8.15 (s, 1H), 7.00–6.93 (m, 1H), 6.63–6.47 (m, 5H), 4.03 (s, 3H), 3.86 (ddd, *J* = 12.8 Hz, *J* = 9.5 Hz, *J* = 2.9 Hz, 1H), 3.49 (ddd, *J* = 13.1 Hz, *J* = 10.1 Hz, *J* = 2.6 Hz, 1H), 3.20–3.01 (m, 4H), 2.85 (ddt, *J* = 13.1 Hz, *J* = 10.4 Hz, *J* = 5.8 Hz, 2H) residues of H2O; ^13^C NMR (100 MHz, CDCl_3_, ppm) δ = 149.8, 141.7, 140.0, 139.6, 139.0, 134.9, 134.1, 133.4, 133.0, 131.7, 130.1, 129.5, 128.4, 62.3, 36.1, 35.0, 34.5, 33.3; EI (m/z, 70 eV, 70 °C): 343/345 (49/49), 312/314 (16/16), 239/241 (23/22), 238/240 (58/62), 208/210 (87/87), 160 (12), 129 (32), 104 (100), 78 (25). HRMS (C_18_H_18_ONBr): Calcd 343.0572, Found 343.0571; IR (ATR, ṽ) = 3063, 3027, 3002, 2983, 2921, 2891, 2849, 2813, 1894, 1594, 1572, 1541, 1497, 1446, 1438, 1424, 1409, 1392, 1333, 1316, 1239, 1200, 1180, 1166, 1119, 1095, 1048, 982, 942, 916, 898, 890, 877, 836, 799, 742, 717, 671, 646, 584, 564, 547, 516, 494, 469, 433, 407, 385 cm^–1^.

Additional information on the chemical synthesis is available via Chemotion repository:

<https://dx.doi.org/10.14272/reaction/SA-FUHFF-UHFFFADPSC-VQEGCJLHLB-UHFFFADPSC-NUHFF-NXICJ-NUHFF-ZZZ>

Additional information on the analysis of the target compound is available via Chemotion repository:

<https://dx.doi.org/10.14272/VQEGCJLHLBTWRF-UDWIEESQSA-N.1>

**(*R*_P_)-1^3^-Bromo-1,4(1,4)-dibenzenacyclohexaphane-1^2^-carbaldehyde** **((*R*_P_)-2)**

Smiles: O=Cc1c2CCc3ccc(CCc(c1Br)cc2)cc3; InChIKey: HOTMMJRJOHGLRS-UHFFFAOYSA-N

The starting material (130 mg, 378 μmol, 1.00 equiv) was dissolved in THF (5.00 mL) and water (0.5 mL). 4-toluenesulfonic acid monohydrate (144 mg, 144 μL, 755 μmol, 2.00 equiv) and formaldehyde (298 mg, 274 μL, 3.8 mmol, 10.0 equiv) were added. The reaction was stirred in a microwave for 4 h at 120 °C, 10 bar and 30 W. The reaction mixture was poured into a flask and the reaction vessel was flushed with THF to collected the material that remained in the reaction vessel. As preparation for the column chromatography (dryload), Celite was added (0.5 g) and the solvent was evaporated. The obtained crude product was purified via flash-chromatography (Interchim, Puriflash, 25 g silica gel) using cyclohexane/ethyl acetate (gradient: 1:0 to 20:1) to obtain 80.6 mg (256 µmol) of the target compound in 68% yield.

*R_f_* = 0.40 (cyclohexane/ethyl acetate 20:1). ^1^H NMR (400 MHz, CDCl_3_, ppm) δ = 10.10 (s, 1H), 6.98 (dd, *J* = 7.9, 1.9 Hz, 1H), 6.68–6.57 (m, 3H), 6.41 (dd, *J* = 7.9 Hz, *J* = 1.9 Hz, 1H), 6.49 (dd, J = 7.9 Hz, *J* = 1.9 Hz, 1H), 3.92 (ddd, *J* = 12.5 Hz, *J* = 9.2 Hz, *J* = 3.0 Hz, 1H), 3.58 (ddd, *J* = 13.3 Hz, *J* = 10.4 Hz, *J* = 2.6 Hz, 1H), 3.24–3.14 (m, 3H), 3.07 (ddd, *J* = 13.2 Hz, *J* = 10.7 Hz, *J* = 2.6 Hz, 1H), 2.88 (ddd, *J* = 14.2 Hz, *J* = 11.0 Hz, *J* = 5.7 Hz, 1H), 2.82 (ddd, *J* = 12.8 Hz, *J* = 10.1 Hz, *J* = 6.9 Hz, 1H). ^13^C NMR (100 MHz, CDCl_3_, ppm) δ = 194.1, 144.8, 140.7, 139.7, 139.0, 138.5, 134.4, 134.1, 133.4, 132.8, 132.0, 131.1, 128.6, 35.3, 34.6, 34.4, 33.2. APCI-MS (*m*/*z*): 315/317 (100/97) [M+1]^+^. HRMS (C_17_H_15_BrO): Calcd 314.0306, Found 315.0374; IR (ATR, ṽ) = 3009, 2939, 2921, 2891, 2874, 2847, 2800, 2766, 1902, 1686, 1594, 1571, 1533, 1497, 1455, 1434, 1407, 1370, 1320, 1261, 1244, 1225, 1193, 1174, 1163, 1118, 1095, 972, 949, 943, 932, 892, 871, 839, 799, 769, 714, 666, 653, 578, 538, 514, 490, 476, 449, 432, 397 cm^–1^.

Additional information on the chemical synthesis is available via Chemotion repository:

<https://dx.doi.org/10.14272/reaction/SA-FUHFF-UHFFFADPSC-HOTMMJRJOH-UHFFFADPSC-NUHFF-NUHFF-NUHFF-ZZZ.1>

Additional analytical information including original data files is available via Chemotion Repository: <https://dx.doi.org/10.14272/HOTMMJRJOHGLRS-UHFFFAOYSA-N.2>

**Origin and Synthesis of compounds described in Fig. 4, 6 and Supplementary Table S2.**

**Supplementary Scheme 3.** Overview of the synthesis of triazole-substituted [2.2]paracyclophanes.

**(rac)-5-(1,4(1,4)-Dibenzenacyclohexaphane-1^2^-yl)-1-phenyl-1*H*-1,2,3-triazole ((rac)-1** ^[29]^

Smiles: c1ccc(cc1)n1nncc1c1cc2ccc1CCc1ccc(CC2)cc1; InChIKey: YNTDRBUGPFYKLY-UHFFFAOYSA-N

Ethylmagnesium bromide solution (287 μL, 861 μmol, 1.00 equiv) was diluted in THF (0.45 mL) and (rac)-1^2^-ethynyl-1,4(1,4)-dibenzenacyclohexaphane (200 mg, 861 μmol, 1.00 equiv) was added to the solution under inert atmosphere. The suspension was heated to 50 °C for 15 min and then allowed to cool to room temperature. Azidobenzene (103 mg, 861 μmol, 1.00 equiv) was added to the reaction mixture and the suspension was vigorously stirred to obtain a dark orange solution. Then the mixture was again heated to 50 °C for 2.5 h, quenched by addition of sat. NH_4_Cl solution and the aqueous phase was extracted with dichloromethane three times. The combined organic layers were concentrated to dryness. The obtained crude product was purified by column chromatography on silica gel (cyclohexane/ ethyl acetate, 9:1 to 4:1) giving 240 mg (683 µmol) of the target compound in 79% yield.

*R_f_* = 0.24 (cyclohexane/ethyl acetate 4:1). ^1^H NMR (400 MHz, CDCl_3_, ppm) δ = 7.95–8.01 (m, 1H), 7.18–7.28 (m, 3H), 7.08–7.14 (m, 2H), 6.59 (dd, *J* = 7.9 Hz, *J* = 1.8 Hz, 1H), 6.50–6.55 (m, 3H), 6.42 (ddd, *J* = 10.5 Hz, *J* = 8.1 Hz, *J* = 1.8 Hz, 2H), 6.28 (d, *J* = 8.3 Hz, 1H), 2.92–3.13 (m, 4H), 2.63–2.75 (m, 2H), 2.32–2.45 (m, 2H); ^13^C NMR (100 MHz, CDCl_3_, ppm) δ = 140.5, 139.4, 139.3, 138.8, 138.3, 136.7, 135.5, 135.0, 133.5, 133.4, 133.2, 132.4, 132.2, 129.4, 128.9 (2C), 128.6, 126.4, 124.4 (2C), 35.3, 35.0, 34.8, 33.4.

Additional information on the chemical synthesis is available via Chemotion repository:

<https://dx.doi.org/10.14272/reaction/SA-FUHFF-UHFFFADPSC-YNTDRBUGPF-UHFFFADPSC-NUHFF-NUHFF-NUHFF-ZZZ.2>

Additional information on the analysis of the target compound is available via Chemotion repository:

<https://dx.doi.org/10.14272/YNTDRBUGPFYKLY-UHFFFAOYSA-N.3>

The compound was synthesized by Martina Austeri during her PhD thesis in the Stefan Bräse Group, Karlsruhe

**(*S*_P_)-5-(1,4(1,4)-Dibenzenacyclohexaphane-1^2^-yl)-1-phenyl-1*H*-1,2,3-triazole ((*S*_P_)-1)**

Smiles: c1ccc(cc1)n1nncc1c1cc2ccc1CCc1ccc(CC2)cc1; InChIKey: YNTDRBUGPFYKLY-UHFFFAOYSA-N

Under a nitrogen atmosphere, ethylmagnesium chloride (5.74 mg, 32.3 μL, 64.6 μmol, 1.00 equiv) was dissolved in 0.5 ml of dry THF in a schlenk tube. (*S*_P_)-1^2^-Ethynyl-1,4(1,4)-dibenzenacyclohexaphane (15.0 mg, 64.6 μmol, 1.00 equiv) was added and due to electrostatic loading the remaining solid was flushed into the solution using 0.3 ml of dry THF. The resulting orange solution was stirred for 15 min at 50 °C then cooled to 21 °C, 30 μl of a previously prepared azidobenzene solution (9.23 mg, 77.5 μmol, 1.20 equiv) was added and the resulting dark orange solution was stirred for another 2.5 h at 50 °C. One more equiv. of ethylmagnesium chloride (5.74 mg, 32.3 μL, 64.6 μmol, 1.00 equiv) was added and the mixture was stirred for another 18 h at 50 °C. After cooling to rt, 5 mL of saturated NH_4_Cl-solution were added, the aqueous phase was extracted with dichloromethane (3 x 10 ml). The combined organic layers were dried over sodium sulfate, filtered and coated onto 200 mg of silica gel. Column chromatography (1 x 13 cm) using cyclohexane/ethl acetate, 10:1 to 4:1 delivered the desired product (3.00 mg, 8.5 μmol, 13% yield).

*R_f_* = 0.24 (cyclohexane/ethyl acetate 4:1). ^1^H NMR (400 MHz, CDCl_3_, ppm) δ = 8.06 (br.s, 1H), 7.36–7.27 (m, 3H), 7.19–7.14 (m, 2H), 6.70–6.56 (m, 4H), 6.54–6.46 (m, 2H), 6.37–6.34 (m, 1H), 3.19–3.03 (m, 4H), 2.81–2.72 (m, 2H), 2.51–2.40 (m, 2H); ^13^C NMR (100 MHz, CDCl_3_, ppm) δ = 140.7, 139.6, 139.5, 139.0, 136.9, 135.6, 135.2, 133.8, 133.5, 133.4, 132.6, 132.4, 129.6, 129.1 (2C), 128.8, 126.7, 124.5 (2C), 110.1, 35.5, 35.2, 34.9, 33.6; EI (m/z, 70 eV, 120 °C): 352 (13) [M+H]^+^, 351 (51) [M]^+^, 323 (20), 220 (17), 219 (90), 218 (100), 217 (23), 204 (16), 191 (10), 116 (15), 115 (41), 105 (27), 104 (59), 77 (15), 57 (14); HRMS (C_24_H_21_N_3_): Calcd 351.1735, Found 351.1735.

Additional information on the chemical synthesis is available via Chemotion repository:

<https://dx.doi.org/10.14272/reaction/SA-FUHFF-UHFFFADPSC-YNTDRBUGPF-UHFFFADPSC-NUHFF-NUHFF-NUHFF-ZZZ>

Additional information on the analysis of the target compound is available via Chemotion repository:

<https://dx.doi.org/10.14272/YNTDRBUGPFYKLY-UHFFFAOYSA-N.1>

**(*R*_P_)-5-(1,4(1,4)-dibenzenacyclohexaphane-1^2^-yl)-1-phenyl-1*H*-1,2,3-triazole ((*R*_P_)-1)**

Smiles: c1ccc(cc1)n1nncc1c1cc2ccc1CCc1ccc(CC2)cc1; InChIKey: YNTDRBUGPFYKLY-UHFFFAOYSA-N

Under a nitrogen atmosphere, ethylmagnesium chloride solution (2M in THF; 25.8 µl, 51.7 µmol, 1.00 equiv) was dissolved in 1 mL of dry THF. (*R*_P_)-1^2^-ethynyl-1,4(1,4)-dibenzenacyclohexaphane (12.0 mg, 51.7 µmol, 1.00 equiv) was added and the mixture was stirred for 30 min at 50 °C. The solution was then cooled to 21 °C, azidobenzene (24.6 mg, 62.0 µmol, 1.20 equiv), predissolved in 120 µl of pentane was added and the reaction was stirred at 50 °C for 22 h. The reaction was cooled to 21 °C, quenched with water and the aqueous layer was extracted with ethyl acetate three times. The combined organic layers were dried over sodium sulfate and coated onto Celite. Column chromatography performed on a glas column 0.5 x 12 cm using CH/EA (4:1) delivered the desired product (2.12 mg, 6.0 μmol, 12% yield).

*R_f_* = 0.23 (cyclohexane/ethyl acetate 4:1). ^1^H NMR (400 MHz, CDCl_3_ [7.27 ppm], ppm) δ = 8.04 (s, 1H), 7.33–7.27 (m, 3H), 7.20–7.16 (m, 2H), 6.67 (dd, *J* = 7.9 Hz, *J* = 1.8 Hz, 1H), 6.64–6.59 (m, 3H), 6.53–6.48 (m, 2H), 6.38–6.35 (m, 1H), 3.18–3.04 (m, 4H), 2.80–2.73 (m, 2H), 2.49–2.42 (m, 2H). The spectrum contains solvent impurities of ethyl acetate (1.27, 2.05, 4.13 ppm), methylene chloride (5.31 ppm) and water (1.56 ppm). The data correspond to the analysis of the racemic compound which is provided here: EI (m/z, 70 eV, 120°C): 352 (13) [M+H]^+^, 351 (47) [M]^+^, 323 (20), 220 (15), 219 (88), 218 (100), 217 (22), 204 (16), 116 (10), 115 (24), 105 (18), 104 (40); IR (ATR, ṽ) = 2922 (m), 2850 (w), 1596 (w), 1496 (s), 1455 (w), 1418 (w), 1256 (w), 1230 (m), 1176 (w), 1091 (w), 1054 (w), 1007 (w), 973 (m), 939 (w), 915 (m), 905 (m), 840 (vs), 795 (w), 761 (vs), 713 (s), 691 (vs), 681 (s), 637 (m), 586 (s), 516 (vs), 490 (m) cm^–1^; HRMS (C_24_H_21_N_3_): Calcd 351.1735, Found 351.1737.

Additional information on the chemical synthesis is available via Chemotion repository:

<https://dx.doi.org/10.14272/reaction/SA-FUHFF-UHFFFADPSC-YNTDRBUGPF-UHFFFADPSC-NUHFF-NUHFF-NUHFF-ZZZ.1>

Additional information on the analysis of the target compound is available via Chemotion repository:

<https://dx.doi.org/10.14272/YNTDRBUGPFYKLY-UHFFFAOYSA-N.2>

**(rac)-5-(1,4(1,4)-Dibenzenacyclohexaphane-1^2^-yl)-1-(4-fluorophenyl)-1*H*-1,2,3-triazole ((rac)-18)**

Smiles: Fc1ccc(cc1)n1nncc1c1cc2ccc1CCc1ccc(CC2)cc1, InChIKey: QHKTTYODSLFKBO-UHFFFAOYSA-N

Ethyl magnesium chloride (76.5 mg, 430 μL, 861 μmol, 2.00 equiv) was dissolved under nitrogen atmosphere in a Schlenk-flask in dry THF (5.00 mL) and (rac)-1^2^-ethynyl-1,4(1,4)-dibenzenacyclohexaphane (100 mg, 430 μmol, 1.00 equiv) was added. The solution was stirred for 15 minutes at 50 °C. The reaction was cooled to room temperature, then 1-azido-4-fluorobenzene (354 mg, 2.6 mmol, 6.00 equiv) was added. The reaction was heated to 50 °C and stirred for 16 hours at this temperature. The reaction mixture was quenched with saturated ammonium chloride solution and the aqueous phase was extracted with methylene chloride. The combined organic phases were dried over Na_2_SO_4_, filtered and the solvent was removed under reduced pressure. The crude product was purified by flash chromatography in cyclohexane:ethyl acetate 4:1 giving 116 mg (313 µmol) of the product in 73% yield.

*R_f_* = 0.28 (cyclohexane/ethyl acetate 4:1). ^1^H NMR (400 MHz, CDCl_3_, ppm) δ = 8.04 (s, 1H), 7.19–7.10 (m, 2H), 7.00–6.91 (m, 2H), 6.65–6.53 (m, 4H), 6.54–6.43 (m, 2H), 6.37–6.34 (m, 1H), 3.16–2.98 (m, 4H), 2.83–2.67 (m, 2H), 2.53–2.38 (m, 2H); ^13^C NMR (100 MHz, ppm) δ = 162.3 (d, *J* = 249.1 Hz), 140.7, 139.5, 139.2, 138.6, 138.4, 135.5, 135.2, 133.5, 133.4, 133.2, 132.8 (d, *J* = 3.1 Hz), 132.5, 132.2, 129.5, 126.2 (d, *J* = 8.7 Hz, 2C), 126.2, 115.9 (d, *J* = 23.1 Hz, 2C), 35.3, 35.0, 34.8, 33.5; ^19^F NMR (376 MHz, CDCl_3_, ppm) δ = –111.97; EI (m/z, 70 eV, 120 °C): 369 (34), 341 (20), 331 (13), 237 (99), 222 (17), 211 (15), 181 (49), 131 (55), 115 (47), 104 (67), 100 (12), 95 (12), 91 (13), 69 (100), 57 (10). HRMS (C_24_H_20_N_3_F): Calcd 369.1641, Found 369.1642; IR (ATR, ṽ) = 3122, 3075, 3033, 3007, 2963, 2924, 2888, 2850, 1898, 1596, 1507, 1479, 1446, 1438, 1419, 1290, 1265, 1218, 1183, 1154, 1136, 1112, 1106, 1095, 1052, 1007, 972, 956, 941, 899, 841, 816, 798, 722, 711, 698, 680, 642, 619, 586, 552, 518, 497, 490, 425, 388 cm^–1^.

Additional information on the chemical synthesis is available via Chemotion repository:

<https://dx.doi.org/10.14272/reaction/SA-FUHFF-UHFFFADPSC-QHKTTYODSL-UHFFFADPSC-NUHFF-NUHFF-NUHFF-ZZZ>

Additional information on the analysis of the target compound is available via Chemotion repository:

<https://dx.doi.org/10.14272/QHKTTYODSLFKBO-UHFFFAOYSA-N.1>

**(*S*_P_)-5-(1,4(1,4)-Dibenzenacyclohexaphane-1^2^-yl)-1-(4-fluorophenyl)-1*H*-1,2,3-triazole ((*S*_P_)-18)**

Smiles: Fc1ccc(cc1)n1nncc1c1cc2ccc1CCc1ccc(CC2)cc1; InChIKey: QHKTTYODSLFKBO-UHFFFAOYSA-N

Ethyl magnesiumchloride (38.2 mg, 215 μL, 430 μmol, 2.00 equiv) was dissolved in dry THF (5.00 mL) under nitrogen atmosphere in a Schlenk flask and (*S*_P_)-1^2^-ethynyl-1,4(1,4)-dibenzenacyclohexaphane (50.0 mg, 215 μmol, 1.00 equiv) was added. The solution was stirred for 15 minutes at 50 °C. The reaction was cooled to room temperature, then 1-azido-4-fluorobenzene (443 mg, 3.2 mmol, 15.0 equiv) was added. The reaction was heated to 50 °C and stirred for 16 hours at this temperature. The reaction mixture was quenched with saturated ammonium chloride solution and the aqueous phase was extracted with methylene chloride. The combined organic phases were dried over Na_2_SO_4_, filtered and the solvent was removed under reduced pressure. The crude product was purified by flash chromatography in cyclohexane:ethyl acetate 4:1 giving 5.0 mg (13.5 µmol) of the product in 6% yield.

*R_f_* = 0.17 (cyclohexane/ethyl acetate 4:1). ^1^H NMR (400 MHz, CDCl_3_, ppm) δ = 8.04 (s, 1H), 7.19–7.12 (m, 2H), 7.03–6.93 (m, 2H), 6.67–6.45 (m, 6H), 6.39–6.36 (m, 1H), 3.19–3.02 (m, 4H), 2.83–2.71 (m, 2H), 2.55–2.38 (m, 2H) residues of water and grease; ^13^C NMR (100 MHz, CDCl_3_, ppm) δ = 162.5 (d, *J* = 249.0 Hz), 140.9, 139.6, 139.4, 138.8, 138.6, 135.7, 135.34, 133.7, 133.6, 133.4, 133.0 (d, *J* = 3.4 Hz), 132.6, 132.4, 129.6, 126.4, 126.4 (d, *J* = 8.6 Hz, 2C), 116.1 (d, *J* = 23.1 Hz, 2C), 35.5, 35.2, 35.0, 33.6; ^19^F NMR (400 MHz, CDCl_3_, ppm) δ = -112.03; EI (m/z, 70 eV, 130 °C): 369 (41), 341 (18), 237 (100), 222 (14), 115 (39), 104 (65). HRMS (C_24_H_20_N_3_F): Calcd 369.1641, Found 369.1642; IR (ATR, ṽ) = 2965, 2927, 2888, 2850, 1605, 1509, 1422, 1414, 1222, 1180, 1153, 1120, 1095, 1086, 1052, 973, 942, 904, 841, 819, 796, 722, 679, 640, 620, 585, 518, 499, 449, 436, 419, 388 cm^–1^.

Additional information on the chemical synthesis is available via Chemotion repository:

<https://dx.doi.org/10.14272/reaction/SA-FUHFF-UHFFFADPSC-QHKTTYODSL-UHFFFADPSC-NUHFF-NUHFF-NUHFF-ZZZ.1>

Additional information on the analysis of the target compound is available via Chemotion repository:

<https://dx.doi.org/10.14272/QHKTTYODSLFKBO-UHFFFAOYSA-N.2>

**(*R*_P_)-5-(1,4(1,4)-Dibenzenacyclohexaphane-1^2^-yl)-1-(4-fluorophenyl)-1*H*-1,2,3-triazole ((*R*_P_)-18)**

Smiles: Fc1ccc(cc1)n1nncc1c1cc2ccc1CCc1ccc(CC2)cc1; InChIKey: QHKTTYODSLFKBO-UHFFFAOYSA-N

Under a nitrogen atmosphere, ethylmagnesium chloride solution (2M in THF; 5.66 mg, 31.9 µL, 63.7 µmol, 1.00 equiv) was dissolved in 0.5 ml of dry THF. (*R*_P_)-1^2^-ethynyl-1,4(1,4)-dibenzenacyclohexaphane (7.40 mg, 31.9 µmol, 1.00 equiv, 0,5 ml of dry THF) was added and the mixture was stirred for 30 min at 50 °C. The solution was then cooled to rt, 1-azido-4-fluorbenzene (34.9 mg, 63.7 µmol, 2.00 equiv) in 80 µl of pentane was added and the reaction was stirred at 50 °C for 22 h. The reaction was cooled to rt, quenched with water and the aqueous layer was extracted with ethyl acetate three times. The combined organic layers were dried over sodium sulfate and coated onto celite. Column chromatography was performed on a glas column 0.5 x 12 cm using cyclohexane/ethyl acetate, 4:1 and delivered the desired product (1.26 mg, 3.41 μmol) in 11% yield.

*R_f_* = 0.25 (cyclohexane/ethyl acetate 4:1). ^1^H NMR (400 MHz, CDCl_3_, ppm) δ = 8.04 (s, 1H), 7.20–7.11 (m, 2H), 7.03–6.93 (m, 2H), 6.67–6.46 (m, 6H), 6.38 (d, *J* = 7.7 Hz, 1H), 3.20–3.03 (m, 4H), 2.85–2.71 (m, 2H), 2.54–2.40 (m, 2H). EI (m/z, 70 eV, 120 °C): 370 (15) [M+H]^+^, 369 (57) [M]^+^, 341 (20), 238 (18), 237 (100), 236 (70), 235 (16), 222 (13), 116 (18), 115 (46), 105 (36), 104 (82), 85 (11), 71 (19), 69 (23), 58 (13), 57 (24); ^19^F NMR (375 MHz, CDCl_3_, ppm) δ = –112.05; HRMS (C_24_H_20_N_3_F): Calcd 369.1641, Found 369.1640.

The data correspond to the analysis of the racemic compound which is provided here: <https://dx.doi.org/10.14272/reaction/SA-FUHFF-UHFFFADPSC-QHKTTYODSL-UHFFFADPSC-NUHFF-NUHFF-NUHFF-ZZZ>.

Additional information on the chemical synthesis is available via Chemotion repository:

<https://dx.doi.org/10.14272/reaction/SA-FUHFF-UHFFFADPSC-QHKTTYODSL-UHFFFADPSC-NUHFF-NUHFF-NUHFF-ZZZ.2>

Additional information on the analysis of the target compound is available via Chemotion repository:

<https://dx.doi.org/10.14272/QHKTTYODSLFKBO-UHFFFAOYSA-N.4>

**(rac)-5-(1,4(1,4)-Dibenzenacyclohexaphane-1^2^-yl)-1-(4-methoxyphenyl)-1*H*-1,2,3-triazole ((rac)-19)**

Smiles: COc1ccc(cc1)n1nncc1c1cc2CCc3ccc(CCc1cc2)cc3; InChIKey: UIUPXPAOUNCBNR-UHFFFAOYSA-N

Ethyl magnesiumchloride (115 mg, 646 μL, 1.3 mmol, 2.00 equiv) was dissolved under nitrogen-atmosphere in a Schlenk flask in dry THF (7.00 mL) and (rac)-1^2^-ethynyl-1,4(1,4)-dibenzenacyclohexaphane (150 mg, 646 μmol, 1.00 equiv) was added. The solution was stirred for 15 minutes at 50 °C. The reaction was cooled to room temperature, then diazonio-(4-methoxyphenyl)azanide (116 mg, 775 μmol, 1.20 equiv) was added. The reaction was heated to 50°C and stirred for 16 hours at this temperature. The reaction mixture was quenched with saturated ammonium chloride solution and the aqueous phase was extracted with methylene chloride. The combined organic phases were dried over Na_2_SO_4_, filtered and the solvent was removed under reduced pressure. The crude product was purified by flash chromatography in cyclohexane:ethyl acetate 4:1 to 2:1 to give 196 mg (512 µmol) of the target compound in 79% yield.

*R_f_* = 0.38 (cyclohexane/ethyl acetate 2:1). ^1^H NMR (400 MHz, CDCl_3_, ppm) δ = 8.01 (s, 1H), 7.11–7.01 (m, 2H), 6.79–6.70 (m, 2H), 6.65–6.52 (m, 4H), 6.52–6.41 (m, 2H), 6.34–6.32 (m, 1H), 3.72 (s, 3H), 3.15–2.95 (m, 4H), 2.73 (td, *J* = 6.1 Hz, *J* = 2.2 Hz, 2H), 2.52–2.40 (m, 2H); ^13^C NMR (100 MHz, CDCl_3_, ppm) δ = 159.5, 140.3, 139.4, 139.3, 138.7, 138.2, 135.4, 134.9, 133.3 (2C), 133.2, 132.4, 132.2, 129.7, 129.4, 126.5, 125.6 (2C), 113.9 (2C), 55.3, 35.3, 35.0, 34.7, 33.5; EI (m/z, 70 eV, 140 °C): 381 (53), 353 (39), 248 (100), 234 (69), 218 (28), 206 (18), 191 (12), 181 (13), 128 (25), 115 (31), 104 (29), 69 (29). HRMS (C_25_H_23_ON_3_): Calcd 381.1841, Found 381.1842; IR (ATR, ṽ) = 3067, 3033, 3012, 2995, 2956, 2929, 2897, 2859, 2837, 2047, 1881, 1608, 1589, 1511, 1463, 1456, 1441, 1422, 1412, 1392, 1312, 1300, 1247, 1228, 1183, 1169, 1159, 1133, 1111, 1088, 1055, 1030, 1018, 1001, 972, 963, 942, 908, 887, 833, 795, 734, 724, 713, 677, 642, 623, 584, 557, 514, 501, 497, 436, 411, 398, 385 cm^–1^.

Additional information on the chemical synthesis is available via Chemotion repository:

<https://dx.doi.org/10.14272/reaction/SA-FUHFF-UHFFFADPSC-UIUPXPAOUN-UHFFFADPSC-NUHFF-NUHFF-NUHFF-ZZZ>

Additional information on the analysis of the target compound is available via Chemotion repository:

<https://dx.doi.org/10.14272/UIUPXPAOUNCBNR-UHFFFAOYSA-N.1>

**(*S*_P_)-5-(1,4(1,4)-Dibenzenacyclohexaphane-1^2^-yl)-1-(4-methoxyphenyl)-1*H*-1,2,3-triazole ((*S*_P_)-19)**

Smiles: COc1ccc(cc1)n1nncc1c1cc2CCc3ccc(CCc1cc2)cc3; InChIKey: UIUPXPAOUNCBNR-UHFFFAOYSA-N

Ethyl magnesiumchloride (38.2 mg, 215 μL, 430 μmol, 2.00 equiv) was dissolved in dry THF (7.00 mL) under nitrogen-atmosphere in a Schlenk-flask and (Sp)-1^2^-ethynyl-1,4(1,4)-dibenzenacyclohexaphane (50.0 mg, 215 μmol, 1.00 equiv) was added. The solution was stirred for 15 minutes at 50 °C. The reaction was cooled to room temperature, then 4-methoxyphenyl azide (48.2 mg, 323 μmol, 1.50 equiv) was added. The reaction was heated to 50 °C and stirred for 16 hours at this temperature. The reaction mixture was quenched with saturated ammonium chloride solution and the aqueous phase was extracted with methylene chloride. The combined organic phases were dried over Na_2_SO_4_, filtered and the solvent was removed under reduced pressure. The crude product was purified by flash chromatography in cyclohexane:ethyl acetate (gradient: 4:1 to 2:1) to give 56.5 mg (148 µmol) of the target compound in 69% yield.

*R_f_* = 0.44 (cyclohexane/ethyl acetate 2:1). ^1^H NMR (400 MHz, CDCl_3_, ppm) δ = 8.02 (s, 1H), 7.11–7.06 (m, 2H), 6.80–6.75 (m, 2H), 6.68–6.45 (m, 6H), 6.37–6.33 (m, 1H), 3.76 (s, 3H), 3.18–3.01 (m, 4H), 2.80–2.72 (m, 2H), 2.52–2.44 (m, 2H) residues of cyclohexane; ^13^C NMR (100 MHz, CDCl_3_, ppm) δ = 159.6, 140.5, 139.5, 139.4, 138.9, 138.4, 135.5, 135.0, 133.5, 133.5, 133.3, 132.5, 132.3, 129.9, 129.5, 126.7 (2C), 125.8, 114.1 (2C), 55.5, 35.4, 35.1, 34.9, 33.6 residues of cyclohexane; MS (FAB, Matrix: 3-NBA), m/z (%): 382 (100), 278 (17), 154 (18), 136 (15), 95 (14). HRMS (C_25_H_24_ON_3_): Calcd 382.1919, Found 382.1918; IR (ATR, ṽ) = 3067, 3003, 2965, 2951, 2927, 2854, 2832, 1612, 1592, 1514, 1476, 1460, 1442, 1435, 1418, 1312, 1300, 1251, 1230, 1207, 1181, 1170, 1156, 1135, 1119, 1111, 1099, 1086, 1054, 1031, 1016, 1001, 972, 958, 938, 919, 907, 884, 829, 792, 724, 710, 684, 676, 643, 619, 585, 552, 523, 511, 490, 465, 429, 414, 375 cm^–1^.

Additional information on the chemical synthesis is available via Chemotion repository:

<https://dx.doi.org/10.14272/reaction/SA-FUHFF-UHFFFADPSC-UIUPXPAOUN-UHFFFADPSC-NUHFF-NUHFF-NUHFF-ZZZ.1>

Additional information on the analysis of the target compound is available via Chemotion repository:

<https://dx.doi.org/10.14272/UIUPXPAOUNCBNR-UHFFFAOYSA-N.2>

**(*R*_P_)-5-(1,4(1,4)-Dibenzenacyclohexaphane-1^2^-yl)-1-(4-methoxyphenyl)-1*H*-1,2,3-triazole ((*R*_P_)-19)**

Smiles: COc1ccc(cc1)n1nncc1c1cc2CCc3ccc(CCc1cc2)cc3; InChIKey: UIUPXPAOUNCBNR-UHFFFAOYSA-N

Under a nitrogen atmosphere ethylmagnesium chloride solution (2M in THF; 7.65 mg, 43.0 μL, 86.1 μmol, 2.11 equiv) was dissolved in 0.5 ml of dry THF, 1^2^-ethynyl-1,4(1,4)-dibenzenacyclohexaphane (9.50 mg, 40.9 μmol, 1.00 equiv. in 0,5 ml of dry THF) was added and the mixture was stirred for 30 min at 50 °C. The solution was then cooled to 21 °C, 4-methoxyphenyl azide (12.8 mg, 86.1 μmol, 2.11 equiv) was added and the reaction was stirred at 50 °C for 22 h. The mixture was cooled to rt, quenched with water and the aqueous layer was extracted with ethyl acetate three times. The combined organic layers were dried over sodium sulfate and coated onto celite. Column chromatography was performed on a glas column 0.5 x 12 cm using cyclohexane/ethyl acetate (4:1) delivering the desired product (6.80 mg, 17.8 μmol) in 44% yield.

*R_f_* = 0.16 (cyclohexane/ethyl acetate 4:1). ^1^H NMR (400 MHz, CDCl_3_, ppm) δ = 8.14 (s, 1H), 7.12–7.05 (m, 2H), 6.83–6.76 (m, 2H), 6.66–6.44 (m, 6H), 6.40–6.36 (m, 1H), 3.79 (s, 3H), 3.18–3.02 (m, 4H), 2.85–2.71 (m, 2H), 2.56–2.39 (m, 2H); ^13^C NMR (100 MHz, CDCl_3_, ppm) δ = 159.7, 140.6, 139.6, 139.5, 139.0, 138.4, 135.6, 135.1, 133.6, 133.5, 133.4, 132.6, 132.4, 129.9, 129.6, 126.7, 125.8 (2C), 114.2 (2C), 55.6, 35.5, 35.2, 34.9, 33.7; IR (ATR, ṽ) = 2921 (m), 2851 (w), 1612 (w), 1592 (w), 1514 (vs), 1462 (m), 1442 (m), 1312 (w), 1300 (w), 1252 (vs), 1230 (s), 1181 (m), 1170 (m), 1111 (w), 1086 (m), 1054 (m), 1031 (s), 1016 (m), 1001 (w), 972 (m), 938 (w), 907 (m), 830 (vs), 792 (s), 724 (m), 710 (m), 676 (m), 643 (m), 620 (m), 585 (vs), 552 (w), 523 (m), 511 (vs), 490 (m), 428 (w), 412 (w) cm^–1^; MS (FAB, Matrix: 3-NBA), m/z (%): 383 [M+H]^+^ (29), 382 [M+] (100), 278 (15), 154 (21), 95 (20), 91 (23); HRMS (C_25_H_24_ON_3_): Calcd 382.1919, Found 382.1920.

Additional information on the chemical synthesis is available via Chemotion repository:

<https://dx.doi.org/10.14272/reaction/SA-FUHFF-UHFFFADPSC-UIUPXPAOUN-UHFFFADPSC-NUHFF-NUHFF-NUHFF-ZZZ.2>

Additional information on the analysis of the target compound is available via Chemotion repository:

<https://dx.doi.org/10.14272/UIUPXPAOUNCBNR-UHFFFAOYSA-N.4>

**(*rac*)-5-(1,4(1,4)-Dibenzenacyclohexaphane-1^2^-yl)-1-(p-tolyl)-1*H*-1,2,3-triazole ((*rac*)-20)**

Smiles: Cc1ccc(cc1)n1nncc1c1cc2ccc1CCc1ccc(CC2)cc1; InChIKey: WQFLLTDMTNWARE-UHFFFAOYSA-N

Ethylmagnesium chloride (76.5 mg, 430 μL, 861 μmol, 2.00 equiv) was dissolved under nitrogen-atmosphere in a Schlenk-flask in dry THF (5.00 mL) and (rac)-1^2^-ethynyl-1,4(1,4)-dibenzenacyclohexaphane (100 mg, 430 μmol, 1.00 equiv) was added. The solution was stirred for 15 minutes at 50 °C. The reaction was cooled to room temperature, then 1-azido-4-methylbenzene (86.0 mg, 646 μmol, 1.50 equiv) was added. The reaction was heated to 50 °C and stirred for 16 hours at this temperature. The reaction mixture was quenched with saturated ammonium chloride solution and the aqueous phase was extracted with CH_2_Cl_2_. The combined organic phases were dried over Na_2_SO_4_ and filtered. As preparation for the column chromatography (dryload), Celite was added (0.3 g) and the solvent was evaporated. After flash-chromatography on silica gel using cyclohexane/ethyl acetate 4:1, 55.7 mg (152 µmol) of the target product was obtained in 35% yield.

*R_f_* = 0.31 (cyclohexane/ethyl acetate 4:1). ^1^H NMR (400 MHz, ppm) δ = 8.03 (s, 1H), 7.11–7.01 (m, 4H), 6.71–6.53 (m, 4H), 6.54–6.44 (m, 2H), 6.37–6.34 (m, 1H), 3.19–3.00 (m, 4H), 2.80–2.72 (m, 2H), 2.52–2.42 (m, 2H), 2.32 (s, 3H); ^13^C NMR (100 MHz, CDCl_3_, ppm) δ = 21.0, 33.5, 34.7, 35.0, 35.3, 124.1 (2C), 126.6, 129.4 (3C), 132.1, 132.4, 133.1, 133.3, 133.4, 134.2, 134.9, 135.4, 138.2, 138.6, 138.7, 139.3, 139.4, 140.4; EI (m/z, 70 eV, 120 °C): 365 (37), 337 (28), 232 (100), 218 (46), 205 (13), 181 (15), 131 (13), 119 (11), 115 (33), 104 (28), 69 (34). HRMS (C_25_H_23_N_3_): Calcd 365.1892, Found 365.1890; IR (ATR, ṽ) = 3135, 3067, 3041, 3033, 3012, 2961, 2928, 2897, 2856, 1592, 1514, 1503, 1473, 1456, 1438, 1422, 1412, 1392, 1380, 1320, 1312, 1282, 1255, 1230, 1205, 1177, 1159, 1133, 1111, 1086, 1052, 1006, 972, 942, 908, 887, 841, 833, 817, 793, 735, 724, 713, 677, 645, 619, 584, 555, 516, 494, 436, 415, 398 cm^–1^.

Additional information on the chemical synthesis is available via Chemotion repository:

<https://dx.doi.org/10.14272/reaction/SA-FUHFF-UHFFFADPSC-WQFLLTDMTN-UHFFFADPSC-NUHFF-NUHFF-NUHFF-ZZZ>

Additional information on the analysis of the target compound is available via Chemotion repository:

<https://dx.doi.org/10.14272/WQFLLTDMTNWARE-UHFFFAOYSA-N.2>

**(*rac*)-5-(1,4(1,4)-dibenzenacyclohexaphane-1^2^-yl)-1-(4-nitrophenyl)-1*H*-1,2,3-triazole ((rac)-21)**

Smiles: [O-][N+](=O)c1ccc(cc1)n1nncc1c1cc2CCc3ccc(CCc1cc2)cc3; InChIKey: UMGYYTZDBFRYCM-UHFFFAOYSA-N

Ethylmagnesium bromide solution (172 mg, 430 μL, 1.29 mmol, 3.00M, 1.00 equiv) was diluted in THF (0.67 mL) and (rac)-1^2^-ethynyl-1,4(1,4)-dibenzenacyclohexaphane hexaphane (300 mg, 1.29 mmol, 1.00 equiv) was added to the solution under Ar atmosphere. The suspension was heated to 50 °C for 15 min and then allowed to cool to room temperature. Azidobenzene (103 mg, 861 μmol, 1.00 equiv, diluted in 0.7 mL of dry THF) was added to the reaction mixture and the suspension was vigorously stirred to obtain a dark orange solution. Then the mixture was again heated to 50 °C for 2.5 h, quenched by addition of a sat NH_4_Cl solution and the aqueous phase was extracted with dichloromethane three times. The combined organic layers were concentrated to dryness. The obtained crude product was purified via flash-chromatography on silica gel using cyclohexane/ethyl acetate 95:5 to 70:30 to give 133 mg (335 µmol) of the target compound in 26% yield.

*R_f_* = 0.53 (cyclohexane/ethyl acetate 2:1). ^1^H NMR (400 MHz, CDCl_3_ [7.27 ppm], ppm) δ = 8.14–8.21 (m, 1H), 8.09 (s, 1H), 7.37–7.44 (m, 2H), 6.65 (td, *J* = 6.9 Hz, *J* = 2.3 Hz, 3H), 6.60 (d, *J* = 1.7 Hz, 1H), 6.54 (dd, *J* = 7.8 Hz, *J* = 1.6 Hz, 1H), 6.50 (dd, *J* = 7.9 Hz, *J* = 1.8 Hz, 1H), 6.41–6.44 (m, 1H), 3.04–3.25 (m, 5H), 2.72–2.88 (m, 2H), 2.35–2.56 (m, 2H). ^13^C NMR (100 MHz, CDCl_3_, ppm) δ = 147.2, 141.4, 141.2, 139.5, 139.1, 138.5, 138.4, 135.8, 135.6, 134.0, 133.4, 133.1, 132.6, 132.2, 129.4, 125.6, 124.6 (2C), 124.5 (2C), 35.3, 35.0, 34.8, 33.4.

Additional information on the chemical synthesis is available via Chemotion repository:

<https://dx.doi.org/10.14272/reaction/SA-FUHFF-UHFFFADPSC-UMGYYTZDBF-UHFFFADPSC-NUHFF-NUHFF-NUHFF-ZZZ>

Additional information on the analysis of the target compound is available via Chemotion repository:

<https://dx.doi.org/10.14272/UMGYYTZDBFRYCM-UHFFFAOYSA-N.1>

The compound was synthesized by Mirja Enders during her PhD thesis in the Stefan Bräse Group, Karlsruhe.

**Origin and synthesis of the used compounds described in Fig. 6 and Supplementary Table S2.**

**Supplementary Scheme 4.** Synthesis of the racemic, (*S*p)-, and (*R*p)-derivatives of precursors **S22**. Compound rac-**S22** was not synthesized (see literature ^28^), it was obtained from the Molecule Archive.

**(rac)-1^2^-Ethynyl-1,4(1,4)-dibenzenacyclohexaphane (rac-S22)**

Compound rac-**S22** was taken as available in the Molecule Archive. The compound was synthesized by Joshua Kramer during his PhD thesis in the Stefan Bräse Group, Karlsruhe. The original syntheses can be retrieved from the literature ^[28]^.

**(*S*_p_)-1^2^-(2,2-dibromovinyl)-1,4(1,4)-dibenzenacyclohexaphane ((*S*_p_)-S21)**

Smiles: BrC(=Cc1cc2CCc3ccc(CCc1cc2)cc3)Br; InChIKey: QTMOJOFFVQKIIZ-UHFFFAOYSA-N

Triphenylphosphane (1.11 g, 4.23 mmol, 2.00 equiv), tetrabromomethane (1.40 g, 4.23 mmol, 2.00 equiv) and zinc (0.277 g, 4.23 mmol, 2.00 equiv) were slowly mixed with dry methylene chloride (10 mL) at 0 °C. After 10 minutes at 0 °C, the ice bath was removed and the reaction was stirred overnight at room temperature. On the following day (*S*p)-1,4(1,4)-dibenzenacyclohexaphane-1^2^-carbaldehyde (*S*_p_)-**S19** (0.500 g, 2.12 mmol, 1.00 equiv) was dissolved in dry methylene chloride (5.0 mL) and added to the reaction mixture. After three hours, 3.5 mL pentane and 1.5 mL methylene chloride were added. The resulting solid was filtered off and washed three times with methylene chloride and pentane. The crude product was purified by flash chromatography in cyclohexane:ethyl acetate 20:1 giving 294 mg (749 µmol) of the target compound in 35% yield.

*R_f_* = 0.77 (cyclohexane/ethyl acetate 4:1). ^1^H NMR (400 MHz, CDCl_3_, ppm) δ = 7.43 (s, 1H), 6.68–6.61 (m, 1H), 6.61–6.50 (m, 4H), 6.51–6.42 (m, 2H), 3.34 (ddd, *J* = 13.2 Hz, *J* = 10.0 Hz, *J* = 2.6 Hz, 1H), 3.19–2.95 (m, 6H), 2.89 (ddd, *J* = 13.6 Hz, *J* = 10.5 Hz, *J* = 5.8 Hz, 1H); ^13^C NMR (100 MHz, CDCl_3_, ppm) δ = 139.9, 139.6, 139.3, 138.4, 136.5, 135.6, 135.0, 133.4, 133.3, 133.2, 133.2, 132.4, 130.2, 90.1, 35.5, 35.2, 34.8, 33.9 residues of cyclohexane; EI (m/z, 70 eV, 80 °C): 392/394 (13/6), 311/313 (32/33), 207/209 (39/38), 128 (80), 104 (100), 78 (11). HRMS (C_18_H_16_Br_2_): Calcd 389.9619, Found 389.9618; IR (ATR, ṽ) = 3006, 2946, 2919, 2887, 2849, 1907, 1595, 1494, 1448, 1432, 1407, 1248, 1203, 1183, 1152, 1108, 1088, 963, 938, 905, 895, 874, 839, 820, 806, 796, 741, 717, 686, 646, 603, 579, 550, 526, 506, 462, 436, 412, 394, 381 cm^–1^.

Additional information on the chemical synthesis is available via Chemotion repository:

<https://dx.doi.org/10.14272/reaction/SA-FUHFF-UHFFFADPSC-QTMOJOFFVQ-UHFFFADPSC-NUHFF-NUHFF-NUHFF-ZZZ>

Additional information on the analysis of the target compound is available via Chemotion repository:

<https://dx.doi.org/10.14272/QTMOJOFFVQKIIZ-UHFFFAOYSA-N.1>

**(*S*_p_)-1^2^-Ethynyl-1,4(1,4)-dibenzenacyclohexaphane ((*S*_p_)-S22)**

Smiles: C#Cc1cc2CCc3ccc(CCc1cc2)cc3; InChIKey: GCRYBHUTYSZNKJ-UHFFFAOYSA-N

The starting material (*S*_P_)-1^2^-(2,2-dibromovinyl)-1,4(1,4)-dibenzenacyclohexaphane (*S*_p_)-**S21** (255 mg, 650 μmol, 1.00 equiv) was dissolved in dry THF (10 mL) under nitrogen-atmosphere in a Schlenk-flask and was cooled to 0 °C. *n*-Butyllithium solution (83.2 mg, 520 μL, 1.3 mmol, 2.00 equiv) was added dropwise and the reaction was stirred for one hour at 0 °C and then for two more hours at room temperature. The reaction mixture was poured on water and the aqueous phase was extracted with methylene chloride. The combined organic phases were dried over Na_2_SO_4_, filtered and the solvent was removed under reduced pressure. The crude product was purified by flash chromatography in cyclohexane:ethyl acetate (gradient: 20:1 to 4:1) giving 130 mg (559 µmol) of the target compound in 86% yield.

*R_f_* = 0.38 (cyclohexane/methylene chloride 4:1). ^1^HNMR (400 MHz, CDCl_3_, ppm) δ = 7.04–6.96 (m, 1H), 6.60–6.42 (m, 6H),3.59 (ddd, *J* = 13.2 Hz, *J* = 10.4 Hz, *J* = 2.8 Hz, 1H), 3.28 (s, 1H), 3.23 (ddd, *J* = 13.0 Hz, *J* = 10.4 Hz, *J* = 5.2 Hz, 1H), 3.15–2.94 (m, 5H), 2.86 (ddd, *J* = 13.1 Hz, *J* = 10.5 Hz, *J* = 5.2 Hz, 1H). ^13^C NMR (100 MHz, CDCl_3_, ppm) δ = 143.1, 139.8, 139.7, 139.5, 137.7, 134.0, 133.5, 133.4, 132.9, 132.7, 130.1, 123.9, 84.1, 80.4, 35.6, 35.2, 34.4, 34.3; EI (m/z, 70 eV, 40 °C): 232 (88), 217 (10), 128 (91), 104 (100), 78 (13). HRMS (C_18_H_16_): Calcd 232.1252, Found 232.1251; IR (ATR, ṽ) = 3303, 3257, 3036, 3010, 2952, 2928, 2918, 2888, 2850, 2772, 2098, 1894, 1588, 1500, 1480, 1449, 1432, 1411, 1402, 1316, 1278, 1239, 1211, 1186, 1153, 1086, 955, 941, 929, 907, 882, 863, 827, 795, 728, 715, 652, 639, 609, 586, 550, 526, 492, 480, 445, 431, 394 cm^–1^.

Additional information on the chemical synthesis is available via Chemotion repository:

<https://dx.doi.org/10.14272/reaction/SA-FUHFF-UHFFFADPSC-GCRYBHUTYS-UHFFFADPSC-NUHFF-NUHFF-NUHFF-ZZZ>

Additional information on the analysis of the target compound is available via Chemotion repository:

<https://dx.doi.org/10.14272/GCRYBHUTYSZNKJ-UHFFFAOYSA-N.1>

**(*R*_p_)-1^2^-Ethynyl-1,4(1,4)-dibenzenacyclohexaphane ((*R*_p_)-S22)**

Smiles: C#Cc1cc2CCc3ccc(CCc1cc2)cc3; InChIKey: GCRYBHUTYSZNKJ-UHFFFAOYSA-N

(*R*_P_)-1^2^-bromo-1,4(1,4)-dibenzenacyclohexaphane (*R*_p_)-**S23** (200 mg, 695 μmol, 1.00 equiv) was weighed into a dried vial. Dichloropalladium;triphenylphosphane (0.122 g, 0.174 mmol, 0.250 equiv), copper(I) iodide (0.0265 g, 0.139 mmol, 0.200 equiv) and triphenylphosphane (0.0730 g, 0.278 mmol, 0.400 equiv) were added, the vial was evaporated and flushed with nitrogen. Then, 5 mL of dry triethylamine and ethynyl(trimethyl)silane (0.478 g, 4.87 mmol, 7.00 equiv) were added, the vial was closed with a crimp cap and shaken at 80 °C for 3 days. After cooling down to 21 °C the crude product was coated onto celite, purified by flash chromatography (50 g biotage cartridge using cyclohexane/dichloromethane 20:1). The solvent was removed under reduced pressure and the obtained product was used in the second step of the reaction without further analysis. In the second step, under an argon atmosphere (*R*_P_)-(1,4(1,4)-dibenzenacyclohexaphane-1^2^-ylethynyl)trimethylsilane (123 mg, 405 μmol, 1.00 equiv) was dissolved in 5 ml of dry THF, tetrabutylazanium;fluoride (0.212 g, 0.811 mmol, 2.00 equiv) was added and the mixture was allowed to stir for 21 h. The crude product was coated onto celite and was purified via flash-chromatography on a glas column (3 x 25 cm) on silica gel using cyclohexane/dichloromethane 20:1. The obtained crude product was purified via flash-chromatography on silica gel using cyclohexane/ethyl acetate 20:1 to 4:1 giving 41.5 mg (0.179 mmol) of the target product in 26% yield.

Rf = 0.27 (Solvent: cyclohexane/dichloromethane, 20:1). ^1^H NMR (400 MHz, CDCl_3_, [7.27 ppm], ppm) δ = 7.02 (d, *J* = 7.8 Hz, 1H), 6.44–6.61 (m, 6H), 3.61 (ddd, *J* = 12.9, 10.5, 2.5 Hz, 1H), 3.30 (s, 1H), 3.21–3.28 (m, 1H), 2.95–3.16 (m, 5H), 2.88 (ddd, *J* = 13.0, 10.6, 5.1 Hz, 1H). ^13^C NMR (100 MHz, CDCl_3_, ppm) δ = 142.9, 139.6, 139.5, 139.3, 137.6, 133.8, 133.3, 133.2, 132.7, 132.5, 129.9, 123.7, 83.9, 80.2, 35.4, 35.1, 34.2, 34.1.

Additional information on the chemical synthesis is available via Chemotion repository:

<https://dx.doi.org/10.14272/reaction/SA-FUHFF-UHFFFADPSC-GCRYBHUTYS-UHFFFADPSC-NUHFF-NUHFF-NUHFF-ZZZ.1>

Additional information on the analysis of the target compound is available via Chemotion repository:

<https://dx.doi.org/10.14272/GCRYBHUTYSZNKJ-UHFFFAOYSA-N.2>

The compound was synthesized by Patricia Kammerer during her master thesis in the Stefan Bräse Group, Karlsruhe

# Supplementary File 2. List of compounds used in this study.

# A separate Excel file provides a list of the compounds used in this study with identifiers of the compounds in the Molecule Archive (KIT-Karlsruhe) allowing other researchers to obtain a stock of the compound for comparison and further studies on request.
