# Supplementary material for: A small molecule screen identifies novel inhibitors of mechanosensory nematocyst discharge in Hydra
Source: Sci Rep. 2021 Oct 18;11:20627. doi: 10.1038/s41598-021-99974-7 (PMC8523708; doi:10.1038/s41598-021-99974-7)
Supplement: Supplementary file 3 — Supplementary Information 3. [file 41598_2021_99974_MOESM3_ESM.pdf]

Supplementary File 3. Custom Python script used for fitting sigmoid curves in Figs. 3, 4, and 6.

```
# -*- coding: utf-8 -*-
"""
Created on Fri May 28 14:31:27 2021

@author: B.Gideon Bergheim
"""
import pandas as pd
import numpy as np
import scipy.optimize as opt
import matplotlib.pyplot as plt

#%% dataset
d = {#2110
     ("2","Y1"):  [100, 100,     None,   70,     None,   40,     40,     None,   None,   0],
     ("2", "Y2"): [100, 100,     100,    70,     50,     50,     40,     30,     20,     0],
     ("2", "Y3"): [100, 100,     70,     80,   70,     None,     30,     50,     30,     0],
     ("2", "Y4"): [None, None,    80,     None,   70,     50,     None,   30,     None,   0],
     ("2", "Y5"): [None, None,    None,   None,   None,   60,     None,   None,   None,   0],
     ("2", "Y6"): [None, None,    None,   None,   None,   None,   None,   None,   20,     0],
     #4599
     ("3","Y1"):  [None, None,    80,     70,     None,   20,     None,   None,   None,   None],
     ("3", "Y2"): [100, 100,     100,    None,   None,   10,     None,   10,     None,   None],
     ("3", "Y3"): [100, 100,     100,    70,     50,     10,     None,   None,   None,   None],
     ("3", "Y4"): [100, 100,     90,     60,     60,     None,   10,     0,      0,      0],
     ("3", "Y5"): [None, None,    None,   None,   50,     None,   20,     None,   0,      0],
     ("3", "Y6"): [None, None,    None,   None,   None,   None,   10,     10,     0,      0],
     #626
     ("1","Y1"): [100, 100,      100,    70,     None,   40,     50,     None,   0,      0],
     ("1", "Y2"): [100, 100,     90,     None,   70,     40,     None,   20,     0,      0],
     ("1", "Y3"): [100, 100,     80,     80,     50,     None,   None,   10,     0,      0],
     ("1", "Y4"): [None, None,    None,   70,     None,   None,   40,     20,     None,   None],
     ("1", "Y5"): [None, None,    None,   None,   70,     60,     20,     None,   None,   None],
     ("1", "Y6"): [None, None,    None,   None,   None,   None,   None,   None,   None,   None]}
df= pd.DataFrame(d, index=(50, 25.0, 12.5,     6.25,   3.13,   1.56,   0.78, 0.39, 0.2, 0.0))
df = df.fillna(value = np.nan)

d2 = {#9928
      ("(Rp)-2","Y1"): [20, 20, 10,    60,     100,    100,    100,    100,    100],
      ("(Rp)-2", "Y2"):[ 0,  10, 30,    50,     80,     100,    100,    100,    100],
      ("(Rp)-2","Y3"): [ 0,  0,  30,    20,     80,     100,    100,    90,     100],
      #7333
      ("(Sp)-2","Y1"): [20, 10, 20,    0,      10,     80,     100,    100,    100],
      ("(Sp)-2","Y2"): [10, 10, 10,    0,      50,     30,     20,     90,    100],
      ("(Sp)-2","Y3"): [ 0,  0,  0,     30,     50,     50,     100,    100,    100]}

df2 = pd.DataFrame(d2, index=( 0.39, 0.78, 1.56, 3.13, 6.25, 12.5, 25, 50, 100))
df2 = df2.fillna(value = np.nan)

d3 = {
      ("X9679","Y1"): [0,   0,  0,  50, 50, 90, 100, 100, 100, 100],
      ("X9679", "Y2"):[0,0,30,30,20,70,90,100,100,100],
      ("X9679","Y3"): [0,0,10,30,50,70,100,100,100,100]}

df3 = pd.DataFrame(d3, index=( 0.2,0.39,0.79,1.56,3.13,6.25,12.5,25,50,100))
df3 = df3.fillna(value = np.nan)

dataset = pd.concat([df,df2,df3],axis=1)
dataset.index.rename("concentration",inplace=True)

d_summary = dataset.T.groupby(level=0).describe().T
d_summary.columns.name = "compound"
means = d_summary .xs("mean",level=1)
std =d_summary .xs("std",level=1)

data = pd.concat([means.stack(), std.stack()],axis=1).reset_index()
data.columns = ['concentration', 'compound', "response", "std"]
data["logConcentration"] = np.log10(data["concentration"])
data = data.replace(-np.inf,np.nan)

data=data.drop(data.loc[data.concentration == 0].index)
c_data = data.groupby("compound")

#%% functions

def sigmoid(x, L ,x0, k, b, rev= False):
```

```
    if rev:
        y = -(np.log((L/x)-b-1))/k+x0
    else:
        y = L / (1 + np.exp(-k*(x-x0)))+b
    return (y)

def format_graph(ax,data):
    plt.legend(loc=7)

    ticks=[1,10,100]
    plt.xticks(np.log10(ticks),ticks, weight= "bold")
    plt.yticks(weight="bold")
    ax.tick_params(axis ='both', which ='both', length = 2)

    plt.ylabel("% inhibition",weight= "bold")
    plt.xlabel("concentration [µM]",weight= "bold")

    plt.ylim(-5,105)

    x=np.linspace(plt.xlim()[0], plt.xlim()[1], 100)
    #line at 50
    ax.plot(x,[50]*len(x), c = "gray",alpha=0.50, markersize=0)
    plt.text(x=plt.xlim()[0]+0.2, y = 51, s= "IC50", color= "gray",alpha=0.50)
    ax.set_facecolor("white")
    # Hide the right and top spines
    ax.spines['right'].set_visible(False)
    ax.spines['top'].set_visible(False)
    ax.spines['bottom'].set_linewidth(2)
    ax.spines['left'].set_linewidth(2)

def create_graph(compound_list,plot_name, d=c_data):
    fig, ax = plt.subplots()
    for name,color in zip(compound_list,["r","g","b"]):
        data = d.get_group(name)
        p0 = [max(data.response), np.median(data.logConcentration),1,min(data.response)]
        popt, pcov = opt.curve_fit(sigmoid, data.logConcentration, data.response,p0, method='dogbox')
        ax.scatter(x=data.logConcentration,y=data.response,c=color)
        x = np.linspace(min(data.logConcentration), max(data.logConcentration), 100)
        y = sigmoid(x,*popt)

        ax.fill_between(x=data.logConcentration, # fill in area of Standard deviation
                        y1=data.response + data["std"],
                        y2=data.response - data["std"],
                        alpha=0.2,
                        color= color,
                        edgecolor=None)

        ax.plot(x,y,"--",linewidth=0.7 ,color= color,label=name)

        #brute force IC50 because the mathmatics did not work
        y=0
        x=0
        while y<50:
            x+=0.001
            y=sigmoid(x,*popt)
        ic50 = round(10**x,2)

        print(name, "force_IC50:", ic50)
        ic502 = sigmoid(50,popt[0],popt[1],popt[2],popt[3], rev= True)
        print(name, "calculated_IC50:", ic502)
        #draw IC50
        plt.vlines(x = x,ymin=0,ymax=50,linewidth=0.50,color="gray",linestyle="--")
        if name == "2":
            x=x-0.20
        plt.text(x=x, y = 1, s= str(ic50), color=color)

    format_graph(ax,data)

    plt.savefig("{}.png".format(plot_name))
    plt.savefig("{}.pdf".format(plot_name))
    plt.savefig("{}.svg".format(plot_name))
#%%
create_graph(["1","2","3"],"compounds")
create_graph(["(Sp)-2","(Rp)-2"],"enantiomeres")
create_graph(["X9679"],"new_protein")
```
